# Effects of biochar-based amendments on enzyme activity, microbial biomass, and bacterial communities in mildly cadmium-contaminated paddy soils

**DOI:** 10.3389/fmicb.2026.1923798

**Published:** 2026-09-03

**Authors:** Junnan Han, Haining Yang, Baowen Huang, Yuan Shi, Wei Peng, Wenjing Zeng, Jian Wang, Donghai Wu, Aike Zhu

**Affiliations:** 1 Nanchong Academy of Agricultural Sciences, Nanchong, Sichuan, China; 2 Key Laboratory of Plant Hormones and Molecular Breeding of Chongqing, School of Life Sciences, Chongqing University, Chongqing, China; 3 Chongqing Haiyu Environmental Protection Technology Co., Ltd., Chongqing, China

**Keywords:** bacterial community, biochar-based amendment, DTPA-Cd, enzyme activity, paddy soils

## Abstract

Mild cadmium (Cd) contamination seriously restricts the safe utilization of paddy soils in southern China. Biochar materials can immobilize soil Cd, yet a single biochar often exhibits limited comprehensive improvement effects. At present, field studies systematically revealing how composite biochar amendments regulate soil enzyme activity, microbial biomass, and bacterial communities remain insufficient. This study aims to clarify the responses of soil enzyme activity, microbial biomass, and bacterial community structure to compound biochar amendments under rice-rape rotation field conditions. We hypothesize that composite amendments consisting of biochar, rape organic fertilizer (ROF), calcium magnesium phosphate fertilizer (CMPF), and fulvic acid (MFA) can optimize soil microhabitat, reduce Cd bioavailability, activate nutrient cycling-related enzymes, and stabilize soil bacterial community diversity. Three different types of biochar, maize straw biochar, bamboo biochar, and coconut shell activated carbon, were mixed with ROF, CMPF, and MFA according to certain proportions, respectively. The results revealed that biochar-based amendments significantly increased urease activity by 19.95%–42.09% (*p* < 0.05); however, acid phosphatase activity was reduced. Additionally, sucrase, nitrate reductase, and cellulase activity did not change significantly compared to the control (CK). Biochar-based amendments significantly increased soil microbial biomass carbon (MBC) and microbial biomass nitrogen (MBN) by 30.10%–64.16 and 12.07%–123.68% (*p* < 0.05), respectively. Maize straw and bamboo biochar amendments significantly increased biomass phosphorus (MBP), but coconut shell activated carbon amendments decreased MBP. Both Chao 1 and Shannon indices of bacterial communities were higher in biochar-based amendments than in CK. *Proteobacteria*, *Chloroflexi*, *Acidobacteriota*, and *Actinobacteriota* dominated across all treatments. Relative abundances of *Proteobacteria* and *Actinobacteriota* slightly increased, whereas *Acidobacteriota* decreased. *Chloroflexi* was enriched by maize straw and bamboo biochar amendments but suppressed by coconut shell activated carbon. At the genus level, biochar-based amendments significantly increased the relative abundance of *Thiobacillus*, with minor influences on other genera. In conclusion, the application of composite biochar-based amendments improves paddy soil quality and promotes the richness and diversity of soil bacterial communities. This study supplements the microecological mechanism of biochar-based amendments technology, offering theoretical support and technical reference for the safe utilization of mildly Cd-polluted paddy soils in Southwest China.

## Graphical abstract

**Figure:**
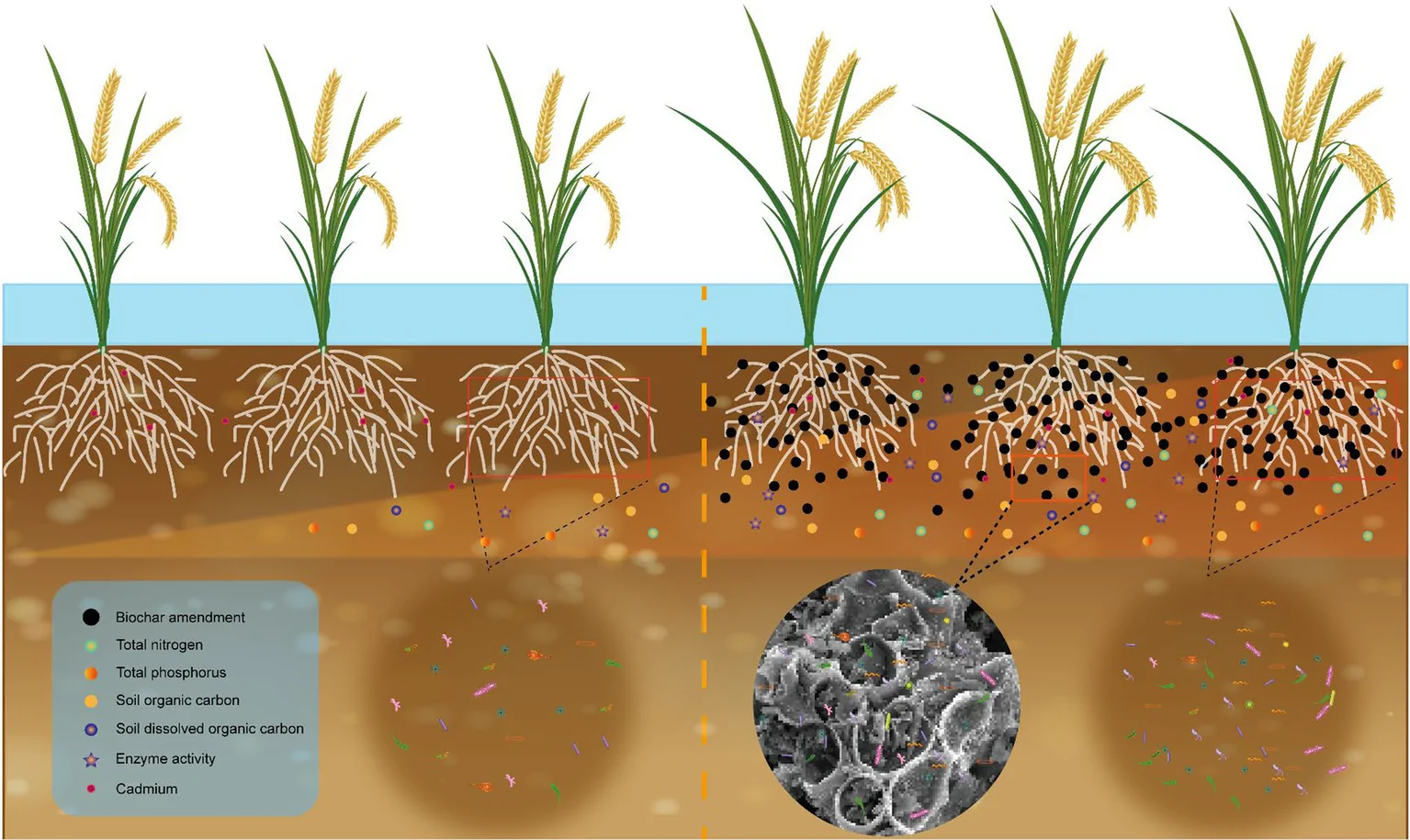

## Highlights

- Bacterial diversity, abundance, and community structures improved with biochar-based amendments.
- Biochar-based amendments increased soil enzyme activity and microbial biomass.
- Soil physicochemical properties and biochar-based amendment characteristics played important roles in altering the network structure of bacterial communities.

## 1 Introduction

Soil heavy metal pollution has become a global environmental problem that threatens agricultural safety and human health. National soil pollution surveys in China have revealed widespread soil contamination, with heavy metal cadmium (Cd) being the most prevalent and hazardous pollutant in farmland soils; 16% of the samples examined had exceeded the National Environmental Quality Standards (NEQS). In particular, soil pollution was serious around mining and industrial activities, and also led to soil contamination in agricultural fields (Huang et al., 2024; Cai et al., 2024; Chen et al., 2024; Yu J. et al., 2024). Cadmium exhibits high mobility and bioavailability in paddy soils, which can be readily enriched in rice grains and transmitted to the human body through the food chain, causing irreversible damage to the liver and kidneys and posing severe carcinogenic risks (Cheng et al., 2023; Pan et al., 2024; Yu L. et al., 2024). *In situ* passivation using biochar and biochar-derived amendment is recognized as a low-cost, environmentally friendly, and efficient technology for remediating mildly Cd-contaminated paddy soils, showing great application prospects in farmland safe utilization (Ma et al., 2023; Zeng et al., 2023; Zhu et al., 2023).

Biochar is a stable, carbon-rich material produced by pyrolysis of biomass at high temperature under oxygen-limited conditions, with prominent soil improvement and carbon sequestration potentials (Zhang et al., 2021). Characterized by pore structure, large specific surface area, strong adsorption capacity, and biodegradation resistance, biochar is primarily composed of amorphous carbon, aromatic hydrocarbons, and ash, with carbon content exceeding 60% (Nan et al., 2022; Xu et al., 2022). Owing to these superior physicochemical properties, biochar and biochar-derived products have been widely applied for the in-situ remediation of heavy metal-contaminated agricultural soils worldwide (Lin et al., 2022; Lv et al., 2021; Samoraj et al., 2022).

Soil enzyme activity, microbial biomass, and community structure are core biological indicators for evaluating soil health, which dominate soil nutrient cycling processes and closely correlate with soil Cd bioavailability and crop growth status (Chen et al., 2020; Cui et al., 2024; Xu et al., 2022; Zhu et al., 2023). Biochar has low viscosity and bulkiness, which can improve soil texture by reducing soil bulkiness and hardness. The carbon skeleton of biochar has excellent water-holding and aeration properties, providing a favorable habitat for microorganisms. Furthermore, the black surface of biochar can absorb more heat to accelerate the growth of microorganisms and increase soil enzyme activity (Yuan et al., 2023). Additionally, applying biochar can directly or indirectly influence soil microbial communities by altering soil physicochemical properties (Singh et al., 2018). Enriched soil nutrients and a suitable environment are prerequisites for the sophistication and sustainability of microbial community networks (Lu et al., 2020). Numerous studies have confirmed that biochar or biochar-derived materials’ application can regulate soil enzyme activity, improve microbial abundance and diversity, and stabilize soil microecological structure by altering soil physicochemical properties. Existing studies have reported the regulatory effects of single biochar materials on soil bacterial communities and enzyme activities in Cd-contaminated soil. Bandara et al. (2022) study showed that the addition of poultry-litter biochar to Cd-contaminated acidic soil decreased the relative abundance of *Firmicutes*, *Chloroflexi*, and *Acidobacteriota*, whereas it increased *Actinobacteriota* and *Proteobacteria*, which they suggested was due to bioaccumulation by increasing soil pH and rehabilitating the soil bacterial communities. Furthermore, Gu et al. (2022) found that biochar effectively enhanced the biodiversity and abundance of microorganisms in sediment as well as reduced Cd and As concentrations. Similarly, Wang et al. (2024) utilized phosphorus-modified biochar to reduce 69.77% of bioavailable Cd, while increasing the sophistication and stabilization of soil microbial communities. Moreover, biochar altered soil enzyme activity. Zheng et al. (2023) study showed that bamboo-derived biochar increased the activity of invertase, urease, and nitrate reductase while decreasing the activity of *Protease*. Luis Moreno et al. (2022) study revealed that biochar significantly reduced soil *β-glucosidase* and protease activities while significantly increasing urease activity. Multiple studies have demonstrated that the application of biochar and biochar-derived materials affected microbial community function and metabolism (Cui et al., 2024; Zhang et al., 2023). Moreover, compound application of biochar with organic and inorganic fertilizers has been proven to achieve better soil improvement and remediation effects than single biochar application (Agegnehu et al., 2016; Chen et al., 2021; Liu et al., 2022; Ndoung et al., 2021; Wang et al., 2023).

Despite extensive research on biochar remediation of Cd-contaminated soil, there remain obvious research gaps that restrict the in-depth understanding and field promotion of this technology. First, most previous studies adopted single biochar materials, while few focused on organic–inorganic composite amendments, which are more adaptable to actual farmland production. Second, most relevant conclusions are derived from laboratory pot incubation experiments, lacking systematic verification under real paddy soils and flooding cultivation conditions. Third, existing studies mostly focus on the macroscopic remediation effects of biochar, including soil Cd passivation efficiency and crop Cd accumulation, while few studies conduct comprehensive multi-index analysis integrating soil enzyme activity, microbial biomass, and high-throughput bacterial community sequencing to reveal microecological regulatory mechanisms. In addition, our previously published study based on the same field trial systematically clarified the physicochemical remediation effect of composite biochar amendments on Cd-contaminated paddy soils and their improvement effect on rice yield and quality (Han et al., 2024). However, this published work only focused on soil Cd immobilization and crop Cd accumulation, ignoring the microecological response mechanism of soil microorganisms and enzymes, which is the key to maintaining the long-term stability of soil remediation effects.

Given the widespread mild Cd contamination of purple paddy soils in Southwest China, single biochar remediation has the defects of high cost and incomplete improvement effect. The composite amendment prepared from local agricultural waste biochar matched with conventional fertilizers has significant economic and ecological promotional value. Nevertheless, the internal microecological mechanism of this compound amendment regulating soil Cd bioavailability and soil health remains unclear, which severely restricts its large-scale popularization and application in farmland remediation. Therefore, it is urgent to carry out field trials to supplement the missing microecological theoretical basis and fill the above research gaps.

Based on the above research gaps and practical application demands, this study further clarified the core research hypothesis and corresponding research objectives. The core hypothesis is that composite amendments can activate enzyme activities, optimize soil microbial biomass characteristics, and stabilize the diversity and structural composition of soil bacterial communities. To verify this hypothesis and fill the existing research deficiencies, a field rice-rape rotation experiment was conducted using three typical agricultural waste biochar materials (maize straw biochar, bamboo biochar, and coconut shell activated carbon) compounded with organic and inorganic additives, with three specific research objectives: (1) To clarify the regulatory effects of different biochar composite formulas on the physicochemical properties and nutrient cycling-related enzyme activities of mildly Cd-contaminated paddy soils; (2) To reveal the response characteristics of soil microbial biomass carbon, nitrogen, phosphorus, and bacterial community diversity and structure to composite biochar amendments under field rotation conditions; (3) To explore the correlation between soil physicochemical properties, DTPA‑Cd bioavailability, and bacterial community succession, and elaborate the underlying mechanisms.

## 2 Materials and methods

### 2.1 Experimental field description

**Figure 1:**
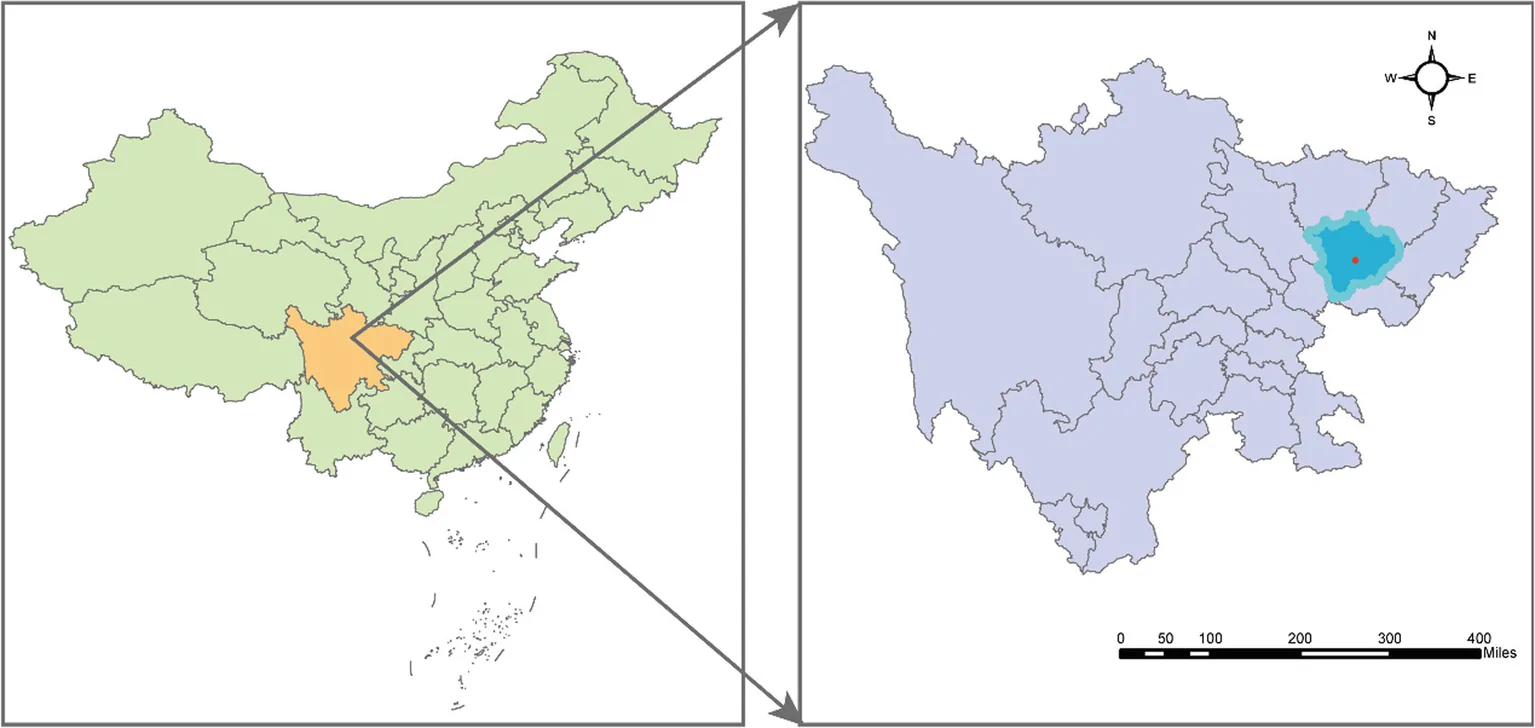
Geographic location of the experimental field site.

The field experiments were conducted in Pengan (31°07′54″N, 106°15′27″E), Nanchong City, Sichuan Province, China (Figure 1). The region is characterized by a mid-subtropical humid monsoon climate with four distinct seasons; the average annual temperature is about 17 °C, annual sunshine duration ranges from 1,200 h to 1,500 h, and the annual rainfall is 1,100 mm. The field soil type is purple sandy loam and is used for rape-rice rotation planting each year. Prior to the field trial, soil samples of 0–20 cm soil layer were collected and tested for physical and chemical properties, including pH of 5.18, soil redox potential (Eh) of 614.2 mV, cation exchange capacity (CEC) of 13.19 cmol kg^−1^, total nitrogen (TN) of 0.96 g kg^−1^, total phosphorus (TP) of 0.24 g kg^−1^, total potassium (TK) of 6.25 g kg^−1^, soil organic carbon (SOC) of 8.84 g kg^−1^, and soil dissolved organic carbon (DOC) of 60.90 mg kg^−1^, total soil Cd (TCd) of 0.32 mg kg^-1,^ and available DTPA-Cd of 0.0741 mg kg^−1^.

### 2.2 Material preparation

The components of biochar-based amendments were biochar, rape organic fertilizer (ROF), calcium magnesium phosphate fertilizer (CMPF), and fulvic acid (MFA). Biochars, CMPF, and MFA were purchased from Henan Haopai Environmental Technology Co., Ltd., Shandong Ruifeng Trading., Ltd., and Jinan Hengyuan Chemical Co., Ltd., Respectively, ROF was purchased at local agricultural markets; the test rice was Fengyouxiangzhan (National authorized rice number 20056) from Nanchong Academy of Agricultural Sciences. Biochars were pyrolyzed using maize straw biochar, bamboo, and coconut shell as feedstock between 500 and 600 °C for 2.5 h under limited oxygen conditions, and the biochar yield was approximately 33.3%. After cooling to room temperature, the biochars were ground into powder by machine and passed through a 200-mesh sieve.

### 2.3 Experimental design

The field experiment was conducted from April to September 2022, using a completely randomized block design with three replicates employed to compare 10 treatments. The specific parameters for each treatment are shown in Table 1. We set up 30 plots, and each plot had an effective plantation area of 20 m^2^ (length × width = 6 m × 3.3 m). Plots were separated with surrounding protective rows of 50 cm width and contain separated irrigation inlets and drainage outlets, and the edges of plots were wrapped in black plastic film to prevent water cascading. Each plot applied an amendment mixture of 20 kg (10 t ha^−1^) 5 d before rice transplanting, and all amendment mixtures were added to the field soil by manual hoeing. Rice was sown on 15 March 2022. Before sowing, rice seeds were disinfected by immersing them in sodium hypochlorite solution (2%) and washed with purified water. Rice was transplanted on 1 May 2022, at a density of 150,000 plants ha^−1^ with rows and plant spacing of 33 cm and 20 cm, respectively. Before rice transplanting, basal compound fertilizer N-P_2_O_5_-K_2_O (23-7-10) was applied at a rate of 810 kg ha^−1^ for all plots to meet rice nutrient demands. In each rice growth stage, field management should follow standard agronomic practices for rice production, such as irrigation, pesticide application, and fertilization. These management measures were applied consistently across all plots.

**Table 1:** Details of experimental treatments.

Treatments	Name	Biochar (%)	ROF^a^ (%)	CMPF^b^ (%)	MFA^c^ (%)
CK	CK	0	0	0	0
A1	A_2_O_6_P_1_F_1_	20	60	10	10
A2	A_4_O_4_P_1_F_1_	40	40	10	10
A3	A_6_O_2_P_1_F_1_	60	20	10	10
B1	B_2_O_6_P_1_F_1_	20	60	10	10
B2	B_4_O_4_P_1_F_1_	40	40	10	10
B3	B_6_O_2_P_1_F_1_	60	20	10	10
C1	C_2_O_6_P_1_F_1_	20	60	10	10
C2	C_4_O_4_P_1_F_1_	40	40	10	10
C3	C_6_O_2_P_1_F_1_	60	20	10	10

A, Maize straw biochar; B, Bamboo biochar; C, Coconut shell activated carbon; ^a^ROF, Rape organic fertilizer; ^b^CMPF, Calcium magnesium phosphate fertilizer; ^c^MFA, Fulvic acid.

### 2.4 Material characterization and soil physicochemical analyses

The microstructure and morphological characteristics of amendment materials were analyzed via scanning electron microscopy coupled with energy-dispersive X-ray spectroscopy (SEM-EDS). Fourier-transform infrared spectroscopy (FTIR) was adopted to characterize surface functional groups. Elemental composition was determined using an elemental analyzer, while specific surface area and pore properties were measured by a Brunauer–Emmett–Teller (BET) surface area and pore size analyzer. Soil samples were collected 3 days before harvest using the five-point sampling method (0–20 cm depth), then divided into two parts: one was air-dried at 20–25 °C, crushed with a wooden hammer, and then sieved through a 2 mm sieve for soil physiochemical properties analyses, and the other was frozen at −80 °C for DNA extraction. Soil pH value was measured with a pH meter (PB-10, Sartorius, Germany) in aqueous suspensions at 1:2.5 soil: water ratios (*w/v*). Soil Eh was measured using Eh meter (PRN-41, DKK-TOA Corporation, Japan). CEC was measured by leaching the soil with ammonium acetate using an exchange-Kjeltec auto analyzer determination directly at pH 7 (Determination of cation exchange capacity of forest soil according to LY/T 1243-1999). Soil particle size was analyzed based on the chemical test method for particle-size analysis of soil (GB/T 27845-2011). TN was determined based on the nitrogen determination methods of forest soil (LY/T 1228-2015). TP was determined using phosphorus determination methods of forest soils (LY/T 1232-2015). TK was determined using the potassium determination methods of forest soils (LY/T 1234-2015). SOC was determined via wet digestion by potassium dichromate. DOC was measured by UP water oscillation extraction-TOC determination using an automatic organic carbon analyzer (Vario TOC cube, Elementar, Germany). For TCd concentrations in soil, soil samples were digested with HNO_3_-HF-HCLO_4_ and then analyzed by inductively coupled plasma emission spectrometer (ICP-OES; Thermo Fisher, USA). The concentrations of DTPA-Cd were extracted with the diethylenetriaminepentaacetic acid (DTPA) extraction method (GB/T 23739-2009) and subsequently were analyzed by ICP-OES.

### 2.5 Soil enzymatic activity analyses

The activity of urease (S-UE) was measured by indophenol blue colorimetry. Fresh soil (2.0 g) was pretreated with toluene, followed by the addition of 10% urea and pH 6.7 citrate buffer, and incubated at 37 °C for 24 h. After dilution and filtration, the filtrate was colored with sodium phenate and sodium hypochlorite, and spectrophotometric detection was conducted at 578 nm. Soil sucrase (S-SC) activity was determined by the 3,5-dinitrosalicylic acid colorimetric method. 2 g of fresh soil sieved through a 10-mesh sieve was mixed with 8% sucrose solution, pH 5.5 phosphate buffer, and toluene, then incubated at 37 °C for 24 h prior to filtration. The filtrate was mixed with 3,5-dinitrosalicylic acid and heated in a boiling water bath for 5 min for color development. After cooling and volume adjustment, spectrophotometric measurement was performed at 508 nm. Soil catalase (S-CAT) activity was measured via volumetric titration. 2 g of fresh soil passing through a 10-mesh sieve was mixed with distilled water and 0.3% H₂O₂, shaken for 20 min, then terminated with sulfuric acid before filtration. 25 mL aliquot of filtrate was titrated with 0.1 N KMnO₄ solution until a pale pink color appeared, with a soil-free blank prepared in parallel. Catalase activity was calculated based on the consumed volume of potassium permanganate. Soil acid phosphatase (S-ACP) activity was measured via colorimetry. 2 g sieved fresh soil was mixed with toluene, pH 6.5 phosphate buffer, and disodium p-nitrophenyl phosphate substrate, followed by incubation at 37 °C for 1 h. After centrifugation, the supernatant was treated with calcium chloride and sodium hydroxide for color development, re-centrifuged at 4,000 r min^−1^, and spectrophotometrically determined at 410 nm with a p-nitrophenol standard curve for quantification. Soil nitrate reductase (S-NR) activity was determined by the phenol disulfonic acid colorimetric method. 1 g fresh soil sieved through a 10-mesh screen was mixed with calcium carbonate, potassium nitrate, and glucose, followed by vacuum pumping and incubation at 30 °C for 24 h. The extract was treated with alum potassium vanadate, and evaporated to dryness, then developed with phenol disulfonic acid. After adjusting to faint yellow with sodium hydroxide and volume fixation, spectrophotometric detection was performed at 400–500 nm. Soil cellulase (S-CL) activity was determined by the 3,5-dinitrosalicylic acid (DNS) colorimetric method. 5 g fresh soil sieved through a 10-mesh screen was mixed with toluene, carboxymethyl cellulose substrate, and pH 5.5 acetate buffer, followed by incubation at 37 °C for 72 h. The filtrate was mixed with the DNS reagent and heated in a boiling water bath for 5 min for color development; after cooling and volume adjustment, spectrophotometric detection was performed at 540 nm.

### 2.6 Microbial biomass determination

The soil microbial biomass carbon (MBC) was determined by the chloroform fumigation extraction method (Wu et al., 1990). The soil microbial biomass nitrogen (MBN) was determined by analyzing the total nitrogen in the extract obtained from fumigation extraction. The concentrations of K_2_SO_4_ extractable-N were determined by Kjeldahl digestion and measured using a calorimetric method (Brookes et al., 1985; Zhu et al., 2017). The microbial biomass phosphorus (MBP) was also analyzed using fumigation extraction methods (Brookes et al., 1982). MBC, MBN, and MBP were calculated by Equations 1–3 as described by Azeem et al. (2023):


$$\mathrm{MBC}=(\begin{array}{l} \text{Extracted}\;C\;\text{fumigated}\\ -\text{Extracted}\;C\;\text{unfumigated} \end{array})\times 2.64$$ (1)

$$\mathrm{MBN}=(\begin{array}{l} \text{Extracted}\;N\;\text{fumigated}\\ -\text{Extracted}\;N\;\text{unfumigated} \end{array})\times 1.00$$ (2)

MBP=(Pfumigated−Punfumigated)×2.50 (3)

### 2.7 DNA extraction and high-throughput sequencing

Total genomic DNA samples were extracted using the OMEGA Soil DNA Kit (M5636-02) (Omega Bio-Tek, Norcross, GA, USA), following the manufacturer’s instructions, and stored at −20 °C prior to further analysis. The concentration and purification of extracted DNAs were measured using a NanaDrop NC2000 spectrophotometer (Thermo Fisher Scientific, Waltham, MA, USA). The quality of DNA was assessed using 2% agarose gel electrophoresis.

PCR amplification of the bacterial 16S rRNA genes V3-V4 region was performed using the forward primer 338F (5′-ACTCCTACGGGAGGCAGCA-3′) and the reverse primer 806R (5′-GGACTACHVGGGTWTCTAAT-3′). Sample-specific 7 bp barcodes were incorporated into the primers for multiplex sequencing. The PCR components contained 5 μL (10 μM) of each Forward and Reverse primer, 1 μL of DNA Polymerase (5 U/μL), 2 μL (2.5 mM) of dNTPs, 1 μL (10 μM) of each Forward and Reverse primer, 1 μL of DNA Template, and 14.75 μL of ddH_2_O. Thermal cycling consisted of initial denaturation at 98 °C for 5 min, followed by 25 cycles consisting of denaturation at 98 °C for 30 s, annealing at 53 °C for 30 s, and extension at 72 °C for 45 s, with a final extension of 5 min at 72 °C. PCR amplicons were purified with Vazyme VAHTSTM DNA Clean Beads (Vazyme, Nanjing, China) and quantified using the Quant-iT PicoGreen dsDNA Assay Kit (Invitrogen, Carlsbad, CA, USA). After the individual quantification step, amplicons were pooled in equal amounts, and pair-end 2 × 250 bp sequencing was performed using the Illumina NovaSeq platform with NovaSeq 6,000 SP Reagent Kit (500 cycles) at Shanghai Personal Biotechnology Co., Ltd. (Shanghai, China). The 16S rRNA gene sequencing results for all samples were submitted to the Sequence Read Archive (SRA) database of the National Centre for Biotechnology Information (NCBI), under the accession code PRJNA1065638.

### 2.8 Processing of Illumina sequencing data

Microbiome bioinformatics was performed with QIIME2 (2019. 4). Raw sequence data were demultiplexed using the demux plugin, followed by primer cutting with the cutadapt plugin (Martin, 2011). Sequences were then quality filtered, denoised, merged, and chimera removed using the DADA2 plugin (Callahan et al., 2016). High-quality sequences were gathered after the chimera was removed for later analysis. The sequences obtained above were merged according to 100% sequence similarity threshold, and the amplicon sequence variants (ASVs) and abundance data were generated. Taxonomy was assigned to ASVs using the classify-sklearn naïve Bayes taxonomy classifier in the feature-classifier plugin against the SILVA Release 132 for bacteria (Bokulich et al., 2018).

### 2.9 Bioinformatics and statistical analysis

Sequence data analyses were mainly performed using QIIME2 and R packages (version 4.3.1). ASV-level alpha diversity indices, such as Chao1 richness, Shannon diversity, Pielou’s evenness, and Good’s coverage, were calculated using the ASV table in QIIME2 and visualized as box plots. ASV-level ranked abundance curves were generated to compare the richness and evenness of ASVs among samples. Beta diversity analysis was performed to investigate the structural variation of microbial communities across samples using Jaccard metrics, Bray-Curtis metrics, and UniFrac distance metrics (Lozupone and Knight, 2005; Lozupone et al., 2007) and visualized via principal coordinate analysis (PCoA), non-metric multidimensional scaling (NMDS), and unweighted pair-group method with arithmetic means (UPGMA) hierarchical clustering (Ramette, 2007). Principal component analysis (PCA) was also conducted based on the genus-level compositional profiles (Ramette, 2007). The significance of differentiation of microbiota structure among groups was assessed by PERMANOVA (Permutational multivariate analysis of variance) (McArdle and Anderson, 2001), ANOSIM (Analysis of similarities) (Warton et al., 2012), and Permdisp (Anderson et al., 2006) using QIIME2. The taxonomy compositions and abundances were visualized using MEGAN (Huson et al., 2011) and GraPhlAn (Asnicar et al., 2015). The Venn diagram was generated to visualize the shared and unique ASVs among samples or groups using the R package “VennDiagram,” based on the occurrence of ASVs across samples/groups regardless of their relative abundance (Zaura et al., 2009). Taxa abundances at the ASV levels were statistically compared among samples or groups by MetagenomeSeq and visualized as Manhattan plots (Zgadzaj et al., 2016). LEfSe (Linear discriminant analysis effect size) was performed to detect differentially abundant taxa across groups using the default parameters (Segata et al., 2011). OPLS-DA (Orthogonal Partial Least Squares Discriminant Analysis) was also introduced as a supervised model to reveal the microbiota variation among groups, using the R package “muma” (Sankar et al., 2008). Random forest analysis was applied to discriminate the samples from different groups using QIIME2 with default settings (Breiman, 2001). Nested stratified k-fold cross-validation was used for automated hyperparameter optimization and sample prediction. The number of k-fold cross-validations was set to 5. Co-occurrence network analysis was performed by SparCC analysis. The pseudocount value in SparCC was set to 10^−6^. The cutoff of correlation coefficients was determined as 70 through random matrix theory-based methods as implemented in the R package RMThreshold. Based on the correlation coefficients, we constructed a co-occurrence network with nodes representing ASVs and edges representing correlations between these ASVs. Network was visualized using the R package igraph and ggraph. Microbial functions were predicted by PICRUSt2 (Phylogenetic investigation of communities by reconstruction of unobserved states) (Douglas et al., 2019) upon MetaCyc^1^ and KEGG^2^ databases.

Environmental factors (pH, CEC, TN, TP, TK, SOC, DOC, TCd, C/N, and DTPA-Cd) were selected by the Variance Inflation Factor (VIF) test. Differences in the microbial composition of the samples can be explained by differences in the explanatory variables. To assess soil environmental factors that influence the microbial community structure, we used redundancy analysis (RDA) for the Bray-Curtis distances of the different soil samples at baseline to perform variance partitioning. Mantel tests and Spearman’s correlation analysis were performed using the R (version 4.3.1) package, and a heatmap of environmental factors and microbial communities was created.

For normally distributed data, which were calculated as the average value of the three replicates, the results were presented as mean ± standard error. The mean and standard deviation were calculated by using Microsoft Excel 2016. One-way ANOVA was performed using IBM SPSS Statistics version 25 (IBM Corp., Armonk, NY, USA) software. Significant differences among treatment groups were evaluated with the least significant difference (LSD) multiple comparisons test at the threshold level of *p* < 0.05. Graphs were generated using the GraphPad Prism 9 (GraphPad Software, San Diego, CA, USA), Rstudio version 1.3.1093, and Adobe Illustrator 2022 (Adobe, San Jose, CA, USA). Pearson’s correlation coefficient (*R*^2^ values) was used for correlation analysis.

## 3 Results

### 3.1 Material characterization and physicochemical properties

**Figure 2:**
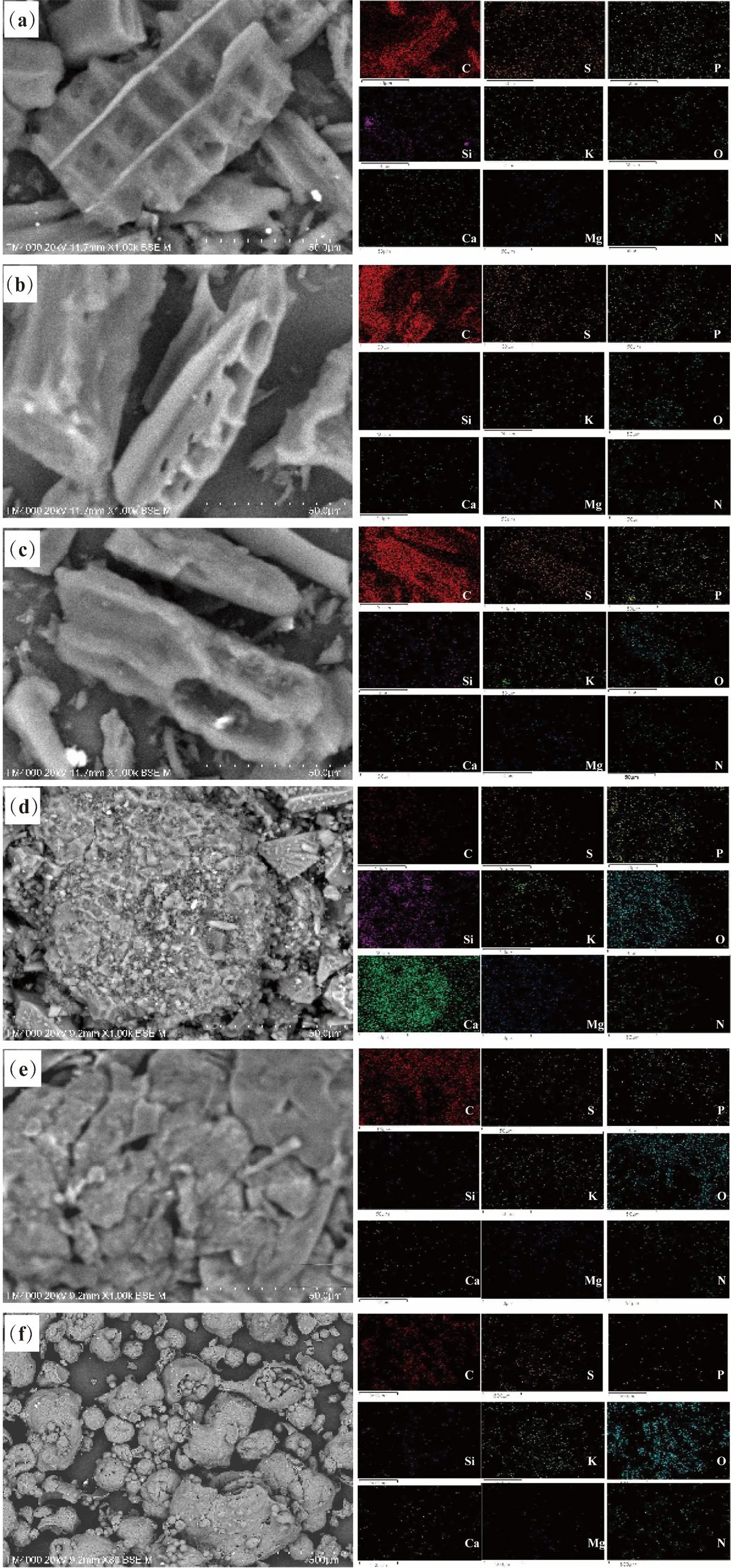
Scanning electron microscope (SEM) images and elemental mapping (mapping) of amendments. **(a)** Maize straw biochar, **(b)** Bamboo biochar, **(c)** Coconut shell activated carbon, **(d)** Calcium magnesium phosphate fertilizer, **(e)** Rape organic fertilizer, **(f)** Fulvic acid.

**Figure 3:**
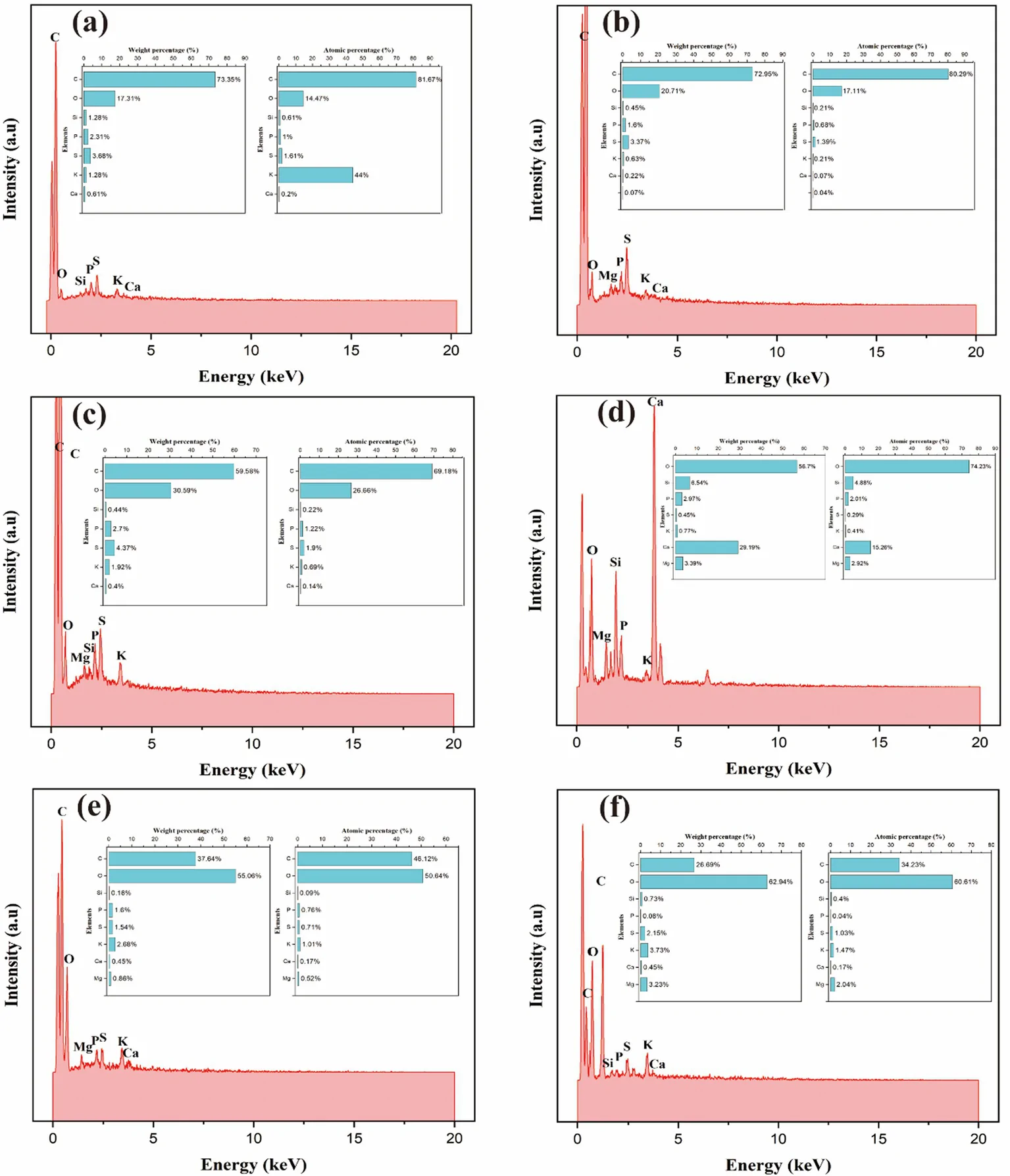
EDS spectra of amendments. **(a)** Maize straw biochar, **(b)** bamboo biochar, **(c)** coconut shell activated carbon, **(d)** calcium magnesium phosphate fertilizer, **(e)** rape organic fertilizer, **(f)** fulvic acid.

Figures 2 and 3 display the micromorphology (SEM), elemental distribution mapping, and energy‑dispersive X‑ray spectroscopy (EDS) of materials. Maize straw biochar (Figure 2a) maintains a hierarchical porous framework generated by volatile release and degradation of cellulose and hemicellulose during pyrolysis, offering ample surface area and transport channels for heavy metal adsorption. Bamboo biochar (Figure 2b) inherits fibrous hollow fissures with highly connected, stable pores; the partially retained layered cell walls serve as physical interception channels and carriers for active sites. Activated coconut shell carbon (Figure 2c) forms a dense micropore-mesopore network. High lignin carbon content and activation etching endow it with higher pore density and specific surface area than biochar. CMPF (Figure 2d) occurs as compact granular aggregates formed by stacking phosphate crystals after calcination. ROF (Figure 2e) consists of organic aggregates mixed with plant residues, microbial metabolites, and minerals, presenting irregular blocks interspersed with pores. MFA (Figure 2f) forms flocculent granular aggregates bound by hydrogen bonds, van der Waals forces, and functional group interactions. Its porous surface provides abundant interfaces for heavy metals adsorption and complexation.

**Figure 4:**
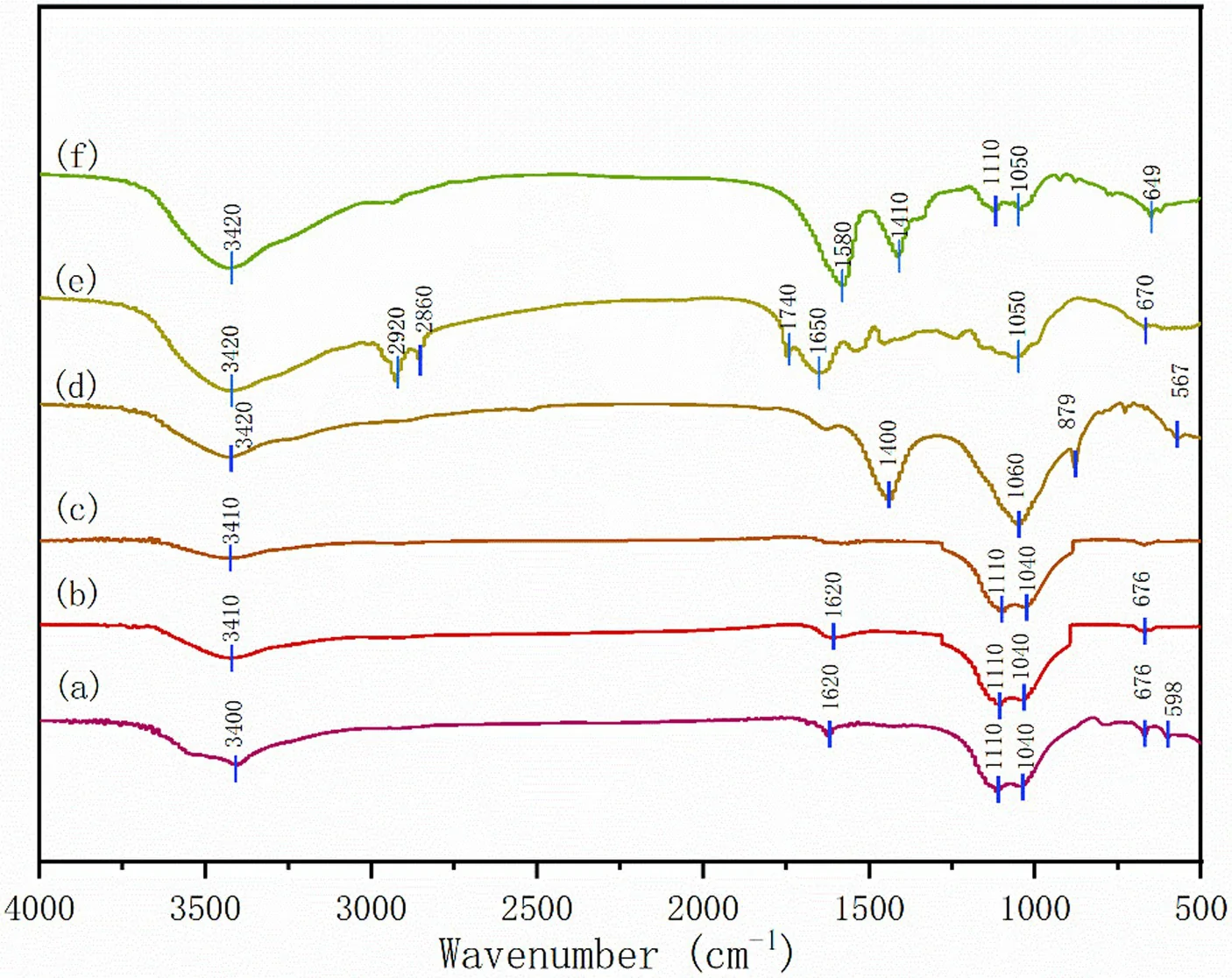
FTIR spectra of amendments: **(a)** Maize straw biochar, **(b)** bamboo biochar, **(c)** coconut shell activated carbon, **(d)** calcium magnesium phosphate fertilizer, **(e)** rape organic fertilizer, **(f)** fulvic acid.

Figure 4 presents the FTIR spectra of amendment materials, with functional groups assigned to each absorption peak listed in Table 2. The band at 3,400–3,420 cm^−1^ (Figures 4a–f) corresponds to stretching vibrations of O-H and N-H, originating from polar phenolic hydroxyl, carboxyl, and amino groups. Absorption at 2,800–3,000 cm^−1^, assigned to aliphatic C-H stretching of methyl and methylene groups, is only detected in ROF (Figure 4e). The peak at 1,600–1,650 cm^−1^ (Figures 4a,b,e) arises from aromatic C=C and C=O stretching. Aromatic structures enable heavy metal adsorption via π-π interactions, while carbonyl groups participate in complexation. The 1,000–1,200 cm^−1^ (Figure 4a–f) region is attributed to C–O (alcohol, ether, ester) and Si–O stretching, reflecting the esterification and etherification of organic functional groups. Bands ranging from 500 to 800 cm^−1^ (Figures 4a,b,d,e,f) are associated with inorganic bonds, including P–O, Ca–O, and Mg–O.

**Table 2:** The FTIR vibration band positions and functional group assignment for amendments.

Amendment materials	Absorption peak position (cm^−1^)	Assigned functional groups
Biochar A	3,400	O–H, N–H
	1,620	C=C, C=O, H–O–H, N–H
	1,110, 1,040	C–O–C, C–C, Si–O–Si
	676, 598	C–H, C–S, C–Br
Biochar B	3,410	O–H, N–H
	1,620	C=C, C=O, H–O–H, N–H
	1,110, 1,040	C–O–C, C–C, Si–O–Si
	676	C–H, C–S, C–Br
Biochar C	3,410	O–H, N–H
	1,110, 1,040	C–O–C, C–C, Si–O–Si
ROF	3,420	O–H, N–H
	2,920, 2,860	C–H, C=O
	1,740, 1,650	C=C, C=O, H–O–H, N–H
	1,050	C–O–C, C–C, Si–O–Si
	670	C–H, C–S, C–Br
CMPF	3,420	O–H
	1,400	C=O, O–H, N–H, P–O
	1,060, 879	Si–O–Si, P–O
MFA	3,420	O–H, N–H
	1,580	C=C, C=O, H–O–H, N–H
	1,410	C=O, C–H, O–H, C–C, N–O
	1,110, 1,050	C–O–C, C–C, Si–O–Si

A, Maize straw biochar; B, Bamboo biochar; C, Coconut shell activated carbon; ^a^ROF, Rape organic fertilizer; ^b^CMPF, Calcium magnesium phosphate fertilizer; ^c^MFA, Fulvic acid.

The elemental composition and molar ratios (H/C, O/C) of different amendment materials are summarized in Table 3. The three biochar samples (Biochar A-C) exhibited high carbon contents ranging from 78.87 to 83.61%, with low H/C (0.18–0.42) and O/C (0.10–0.12) molar ratios. The H/C molar ratio is an indicator of aromaticity, where lower values correspond to higher aromaticity. The O/C molar ratio reflects the abundance of oxygen-containing functional groups and hydrophobic-hydrophilic properties; lower O/C values indicate stronger hydrophobicity. Accordingly, these biochars exhibited high pyrolytic carbonization degrees, well-developed aromatic frameworks, and strong hydrophobicity. ROF displayed relatively low C content (36.31%), along with elevated H, N, and O fractions, corresponding to much higher H/C (2.53) and O/C (0.78) values. As an inorganic phosphate-based material, CMPF possessed extremely low C, H, and N contents. MFA showed abundant O (52.61%) and moderate C (34.23%), yielding high H/C (1.63) and O/C (1.31) molar ratios, which reflected its abundant oxygen-containing functional groups.

**Table 3:** Main element composition of amendments.

Amendment materials	C (%)	H (%)	N (%)	O (%)	S (%)	H/C (molar ratio)	O/C (molar ratio)
Biochar A	78.87	2.16	0.73	12.59	0.25	0.33	0.12
Biochar B	80.70	2.85	0.42	10.89	0.17	0.42	0.10
Biochar C	83.61	1.27	0.30	11.67	0.23	0.18	0.10
ROF	36.31	7.65	6.20	37.88	1.12	2.53	0.78
CMPF	2.53	0.00	0.00	38.90	0.15	0.00	11.53
MFA	34.23	4.11	2.07	52.61	1.03	1.63	1.31

A, Maize straw biochar; B, Bamboo biochar; C, Coconut shell activated carbon; ^a^ROF, Rape organic fertilizer; ^b^CMPF, Calcium magnesium phosphate fertilizer; ^c^MFA, Fulvic acid.

Table 4 shows the materials’ physicochemical properties. Biochars and CMPF were alkaline, while ROF and MFA were weakly acidic. Maize biochar exhibited strong alkalinity (pH > 10). All biochars had high CEC values (>20 cmol kg^−1^) and higher organic carbon contents than organic fertilizer and MFA, indicating prominent carbon sequestration capacity. MFA and CMPF contained low heavy metal levels, whereas biochars and organic fertilizer showed relatively higher heavy metal concentrations due to pyrolytic enrichment of straw-derived metals. Biochar ash content decreased in the order: maize biochar > coconut shell activated carbon > bamboo biochar, because inorganic minerals remained after pyrolysis. Coconut shell activated carbon and bamboo biochar had high iodine adsorption capacity owing to developed micropores, while no iodine adsorption was detected for organic fertilizer, CMPF, and MFA. Coconut shell activated carbon, bamboo biochar, and MFA exhibited large specific surface areas, attributed to rich pore structures and flocculent aggregation. By contrast, CMPF had an extremely low specific surface area due to its dense non-porous crystalline structure.

**Table 4:** Detailed physiochemical parameters of the amendments.

Physiochemical parameters	Biochar A	Biochar B	Biochar C	ROF	CMPF	MFA
pH	10.4	8.2	8.0	6.1	8.5	6.7
CEC (cmol kg^−1^)	25.8	27.8	26.2	5.8	ND^a^	8.92
TCd (μg kg^−1^)	6.04	5.01	5.99	15.00	6.85	1.25
TPb (μg kg^−1^)	128	96	136	108	65	1.27
TAs (μg kg^−1^)	25	23	36	21	25	1.00
TCr (μg kg^−1^)	500	368	566	426	180	8.99
Ash content (%)	11.2	5.8	8.1	ND	ND	ND
Iodine adsorption (mg g^−1^)	706	750	1,012	ND	ND	ND
BET (m^2^ g^−1^)	40.2	80.6	95.6	45.6	2.1	159.6

A, Maize straw biochar; B, Bamboo biochar; C, Coconut shell activated carbon; ^a^ROF, Rape organic fertilizer; ^b^CMPF, Calcium magnesium phosphate fertilizer; ^c^MFA, Fulvic acid; ^a^ND, not detected.

### 3.2 Soil physiochemical properties

The physicochemical characteristics of soil for each treatment are presented in Table 5. The amendments increased the soil pH, TN, TP, CEC, SOC, and DOC, as well as decreased the soil Eh in comparison with CK, but had a non-significant effect on soil TP, and had a little effect on soil C/N. The amendments increased soil pH at a range of 0.03–0.39 units. B3, A3, and B2 showed a significant rise in soil pH (*p* < 0.05). B3, B2, A3, A2, and C3 significantly reduced soil Eh by 20.8, 17.7, 16.6, 15.7, and 13.8% (*p* < 0.05). Compared with CK, TN and TP contents were significantly increased except for the A1 treatment (*p* < 0.05). Soil CEC was markedly improved in the range of 21.1% (C2) to 98.3% (B3). SOC and DOC had also been significantly enhanced (*p* < 0.05). The amendments reduced soil DTPA-Cd in the range of 9.58–27.10%. Compared to CK, C1, A1, B1, and B3 treatments significantly reduced soil DTPA-Cd (*p* < 0.05).

**Table 5:** Effect of amendments on soil physiochemical properties.

Treatment	pH	Eh (mV)	TN (g/kg)	TP (g/kg)	TK (g/kg)	CEC (cmol/kg)	SOC (g/kg)	DOC (mg/kg)	DTPA-Cd (mg/kg)	C/N
CK	5.20 ± 0.11c	603.27 ± 11.78a	0.980 ± 0.022c	0.230 ± 0.024c	6.226 ± 0.313a	14.56 ± 4.37d	8.88 ± 0.36c	63.21 ± 5.14c	0.0706 ± 0.0017a	9.07 ± 0.50ab
A1	5.27 ± 0.06bc	558.67 ± 18.08ab	1.035 ± 0.047c	0.243 ± 0.010bc	6.464 ± 0.382a	18.97 ± 1.94 cd	9.15 ± 0.17bc	68.79 ± 1.76bc	0.0515 ± 0.0078b	8.85 ± 0.34abc
A2	5.42 ± 0.10abc	508.60 ± 33.65bc	1.117 ± 0.019b	0.266 ± 0.015abc	6.655 ± 0.452a	23.72 ± 3.71bcd	9.45 ± 0.50bc	72.32 ± 10.96bc	0.0582 ± 0.0036ab	8.47 ± 0.57bc
A3	5.57 ± 0.11a	503.23 ± 30.01bc	1.133 ± 0.025b	0.276 ± 0.011ab	6.643 ± 0.355a	26.30 ± 1.57abc	10.01 ± 0.74bc	78.49 ± 3.04b	0.0542 ± 0.0125ab	8.84 ± 0.68abc
B1	5.28 ± 0.06bc	545.70 ± 22.71ab	1.134 ± 0.026b	0.278 ± 0.010ab	6.481 ± 0.272a	20.41 ± 3.24bcd	9.40 ± 0.65bc	72.87 ± 7.71bc	0.0521 ± 0.0043b	8.29 ± 0.41bc
B2	5.44 ± 0.17ab	496.37 ± 54.68bc	1.126 ± 0.031b	0.280 ± 0.018ab	6.545 ± 0.336a	25.22 ± 2.59ab	9.63 ± 0.50bc	75.05 ± 9.99b	0.0550 ± 0.0057ab	8.55 ± 0.22bc
B3	5.59 ± 0.15a	477.57 ± 14.19c	1.157 ± 0.021ab	0.270 ± 0.012abc	6.686 ± 0.298a	28.87 ± 0.84a	9.96 ± 0.38bc	80.34 ± 4.52b	0.0536 ± 0.0022b	8.61 ± 0.34bc
C1	5.31 ± 0.16bc	561.70 ± 36.37ab	1.153 ± 0.034ab	0.281 ± 0.008ab	6.643 ± 0.387a	19.00 ± 2.45 cd	9.34 ± 0.66bc	74.87 ± 1.90b	0.0515 ± 0.0148b	8.09 ± 0.37c
C2	5.26 ± 0.15bc	547.60 ± 54.95ab	1.165 ± 0.097ab	0.293 ± 0.049a	6.569 ± 0.123a	17.63 ± 0.56d	10.38 ± 1.28b	76.71 ± 5.41b	0.0597 ± 0.0149ab	8.89 ± 0.41abc
C3	5.23 ± 0.06bc	520.30 ± 42.04bc	1.228 ± 0.023a	0.305 ± 0.038a	6.631 ± 0.773a	20.92 ± 4.37bcd	11.69 ± 0.41a	96.90 ± 2.43a	0.0638 ± 0.0048ab	9.52 ± 0.33a

Data are presented as mean ± standard error (*n* = 3). Same lowercase letters mean no significant difference while different lowercase letters mean significant differences between groups (ANOVA, LSD test, *p* < 0.05).

### 3.3 Effect of biochar-based amendment on soil enzyme activity and soil microbial biomass

**Figure 5:**
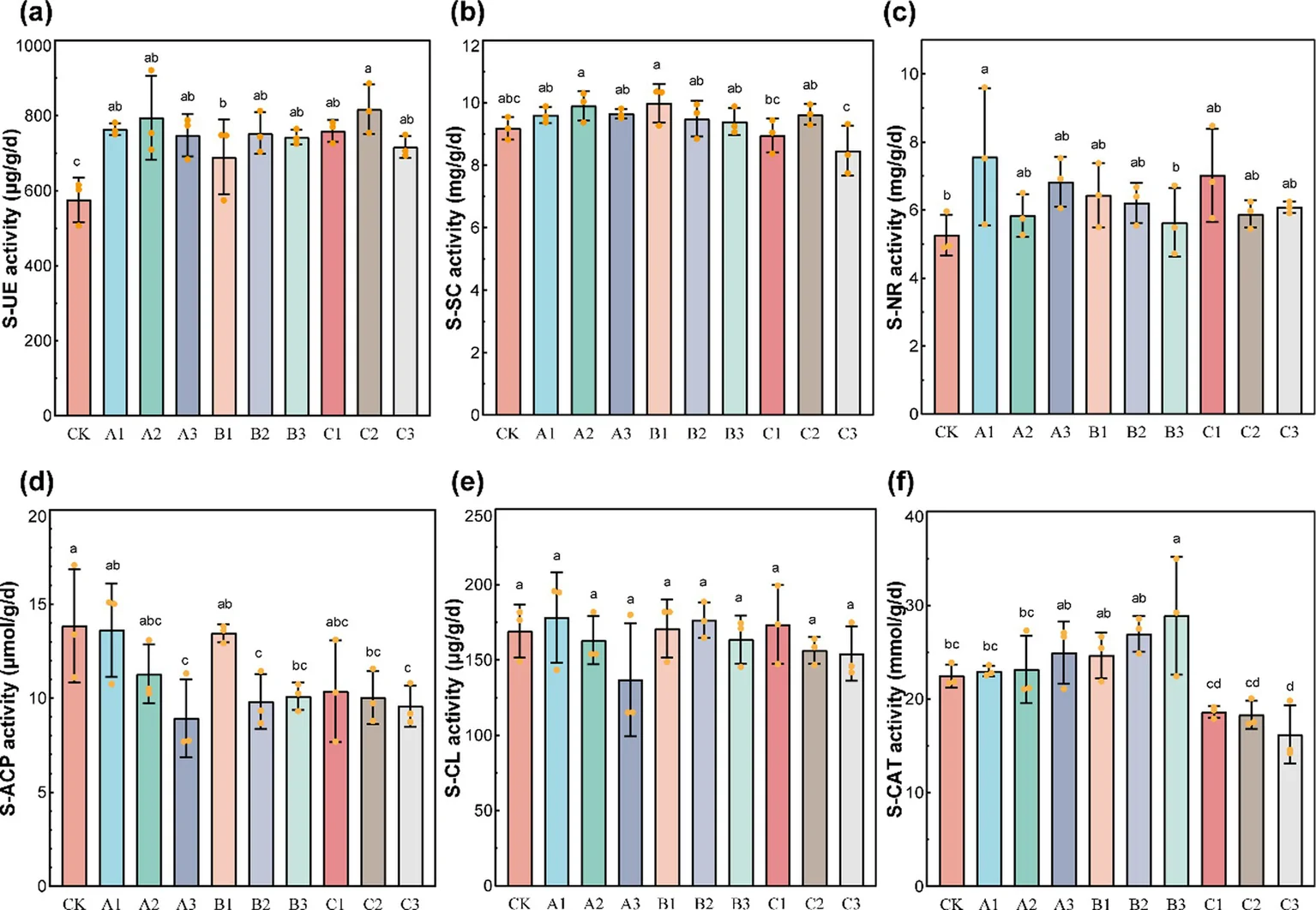
Effect of amendments on the activities of **(a)** urease (S-UE), **(b)** sucrase (S-SC), **(c)** nitrate reductase (S-NR), **(d)** acid phosphatase (S-ACP), **(e)** cellulase (S-CL), and **(f)** catalase (S-CAT). Error bars represent the standard deviation of triplicates. Data are presented as mean ± standard error (*n* = 3). The same lowercase letters mean no significant difference while different lowercase letters mean significant differences between groups (ANOVA, LSD test, *p* < 0.05).

Soil enzyme activities were used to evaluate the soil functions as shown in Figure 5, which illustrates the variation of soil enzyme activities in S-UE, S-SC, S-NR, S-ACP, S-CL, and S-CAT. The S-UE activities in all treatments were significantly increased compared to CK (*p* < 0.05), and the increase of S-UE activities was in the range of 19.95%–42.09% (Figure 5a). As shown in Figures 5b,c,e, the S-SC activities, S-NR activities, and S-CL activities were slightly less dynamic and were insignificant compared to CK (*p* < 0.05). The S-ACP activities of all the treatments were decreased (Figure 5d). A3, C3, B2, C2, and B3 significantly reduced S-ACP activities by 35.61, 30.87, 29.15, 27.56, and 27.10% (*p* < 0.05), respectively. Compared with the CK (Figure 5f), B2, A3, B1, A2, and A1 treatments enhanced S-CAT activities, whereas B3 treatment showed significantly increased S-CAT activities by 28.47% (*p* < 0.05), whereas C3, C2, and C1 decreased S-CAT activities by 27.86, 18.47, and 17.19%, respectively.

**Figure 6:**
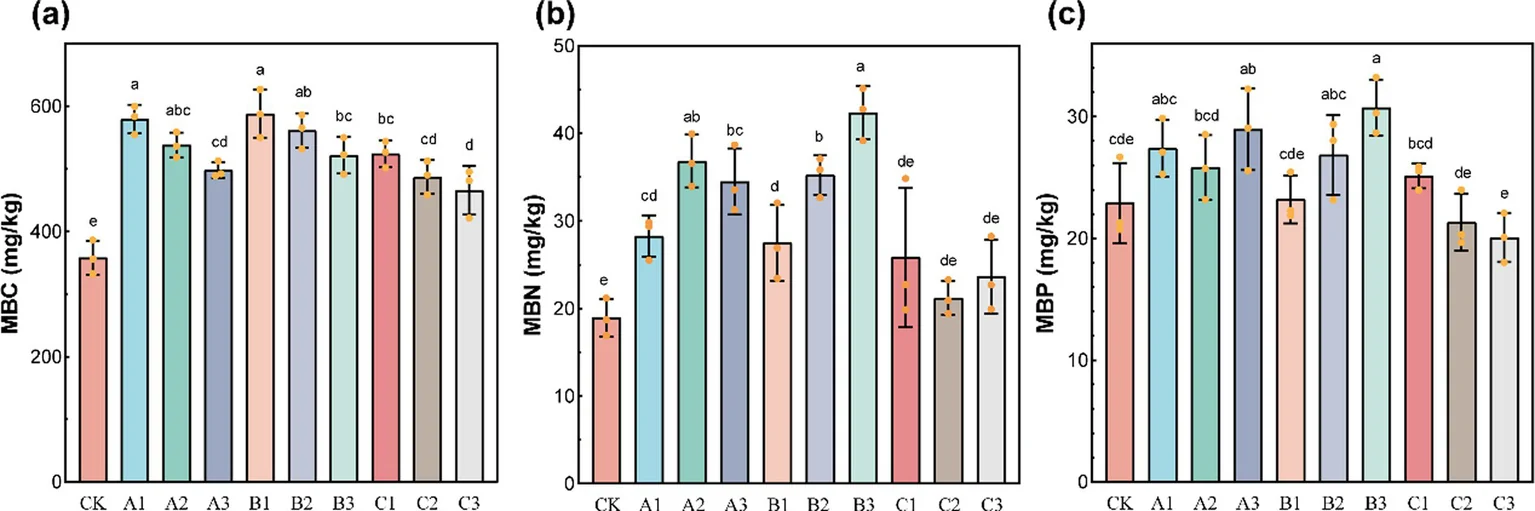
Effect of amendments on soil **(a)** microbial biomass carbon (MBC), **(b)** microbial biomass nitrogen (MBN), and **(c)** microbial biomass phosphorus (MBP). Error bars represent the standard deviation of triplicates. Data are presented as mean ± standard error (*n* = 3). The same lowercase letters mean no significant difference, while different lowercase letters mean significant differences between groups (ANOVA, LSD test, *p* < 0.05).

Soil microbial biomass is the active nutrient reservoir in the soil and is an important bioindicator of soil fertility and quality. According to Figure 6a, compared with CK, all the treatments significantly increased soil MBC (*p* < 0.05), and the increments of soil MBC were in the range of 30.10–64.16%. Figure 6b presented that all amendments increased soil MBN, B3, A2, B2, A3, A1, and B1 significantly improved soil MBN by 123.68, 94.55, 43.55, 85.93, 82.24, 49.16, and 45.13% (*p* < 0.05), respectively, compared with CK. Furthermore, as shown in Figure 6c, B3 and A3 significantly increased soil MBP by 34.13 and 26.44% (*p* < 0.05), followed by A1 (19.67%), B2 (17.23%), A2 (12.72%), C1 (9.69%), and B1 (1.29%), nevertheless, C3 and C2 were decreased soil MBP by 12.40 and 6.94% (*p* < 0.05).

### 3.4 Sequence data and bacterial community diversity

Sequenced 30 samples, a total of 2,326,404 sequences were obtained, an average of 77,547 sequences per sample, the distribution of sequence length ranged from 230 bp to 443 bp, and the average sequence length was 417.98 bp. After filtering, denoising, merging, and non-chimeric processing, 1,249,595 high-quality sequences were ultimately achieved (Supplementary Table S1), which accounted for 53.71% of the total, and the obtained high-quality sequences were used for downstream analysis. As shown in Supplementary Table S2, Figure S1, which were notes on taxonomy species and statistics on the number of classified units, the maximum number of ASVs annotated was in the genus level and the minimum at the domain level, and the taxonomic annotations species of all treatments ranged from 304.52 to 396.95 on average for the domain, phylum, class, order, family, genus, and species.

**Figure 7:**
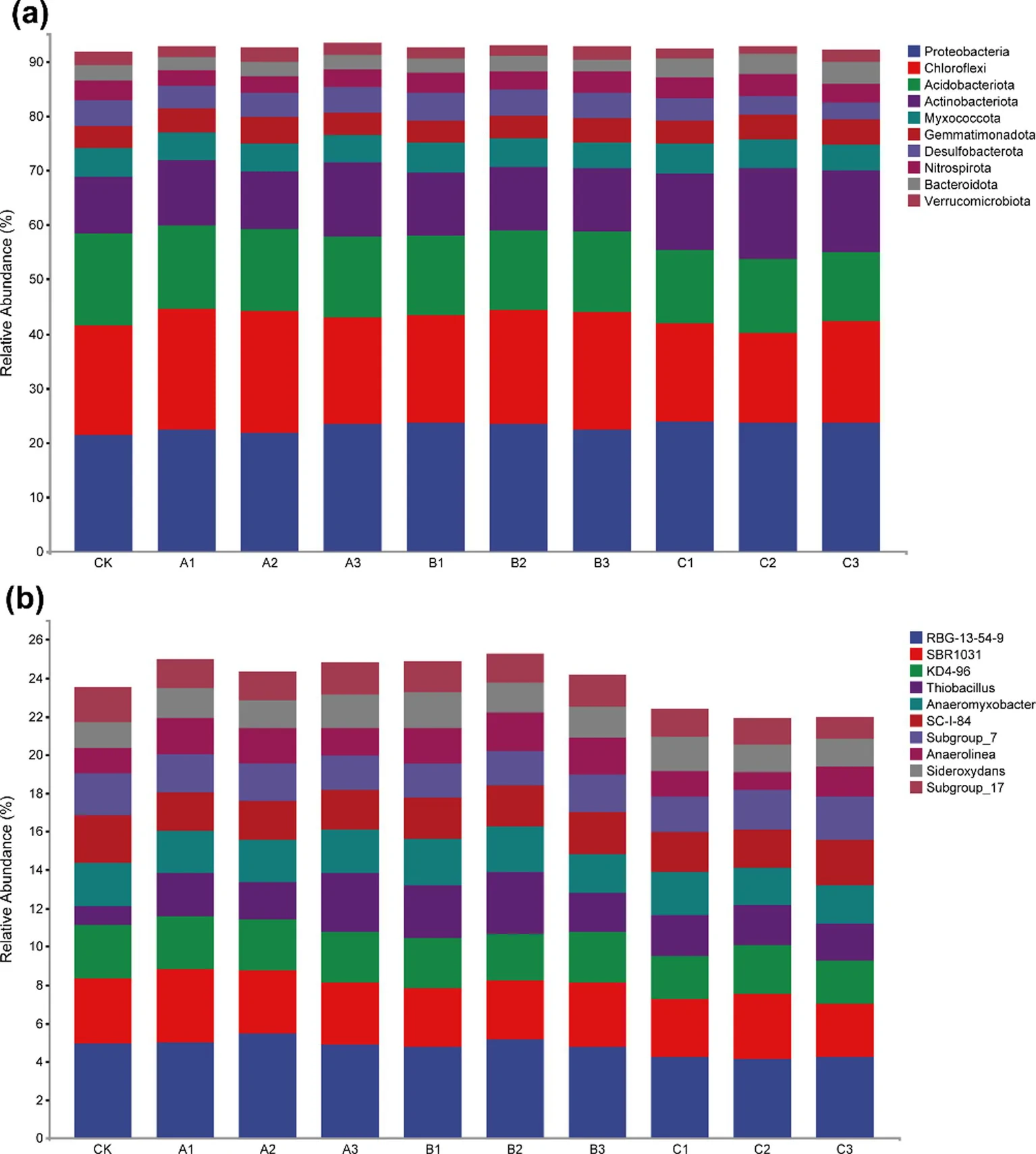
Relative abundance of bacterial communities at phylum **(a)** and genus **(b)** species levels during biochar-based soil amendment.

As illustrated in Supplementary Table S3, the number of microbial taxonomic units at the level of phylum and genus showed a decreasing trend in all treatments compared to the CK. The top 10 dominant phyla of bacterial communities in each sample were *Proteobacteria*, *Chloroflexi*, *Acidobacteriota*, *Actinobacteriota*, *Myxococcota*, *Gemmatimonadota*, *Desulfobacterota*, *Nitrospirota*, *Bacteroidota*, and *Verrucomicrobiota,* with the relative abundances accounting for 19.49%–26.03, 15.43%–27.01, 11.64%–18.71, 9.31%–19.02, 4.20%–5.92, 3.17%–5.39, 2.72%–6.10, 1.90%–5.09, 2.05%–4.47, and 0.70%–4.00%, respectively (Figure 7), together constituting over 75%. As shown in Figure 7a, Supplementary Table S4, in comparison to CK, all treatments reduced the relative abundance of *Acidobacteriota*, while C3, C2, and C1 significantly reduced the relative abundance of *Acidobacteriota* (*p* < 0.05), the decreased from 16.94 to 12.69%. All treatments increased the relative abundance of *Actinobacteriota* compared to CK, with A3, C1, C2, and C3 significantly increasing the abundance of *Actinobacteriota* (*p* < 0.05), the increased from 10.32 to 16.74%. C3 treatment significantly decreased the relative abundance of *Desulfobacterota* (*p* < 0.05), while C3 and C2 significantly increased the relative abundance of *Bacteroidota* (*p* < 0.05). However, there were insignificant effects of the top 10 dominant genera of bacterial communities in each treatment, in addition to *Thiobacillus* (Figure 7b; Supplementary Table S5), compared with CK, A3, B1, and B2 treatments, which significantly increased the relative abundance of *Thiobacillus* (*p* < 0.05). As Supplementary Figure S2 shows more visually and clearly, the portions of each treatment in each dominant bacterium.

### 3.5 Alpha diversity of the bacterial community

**Figure 8:**
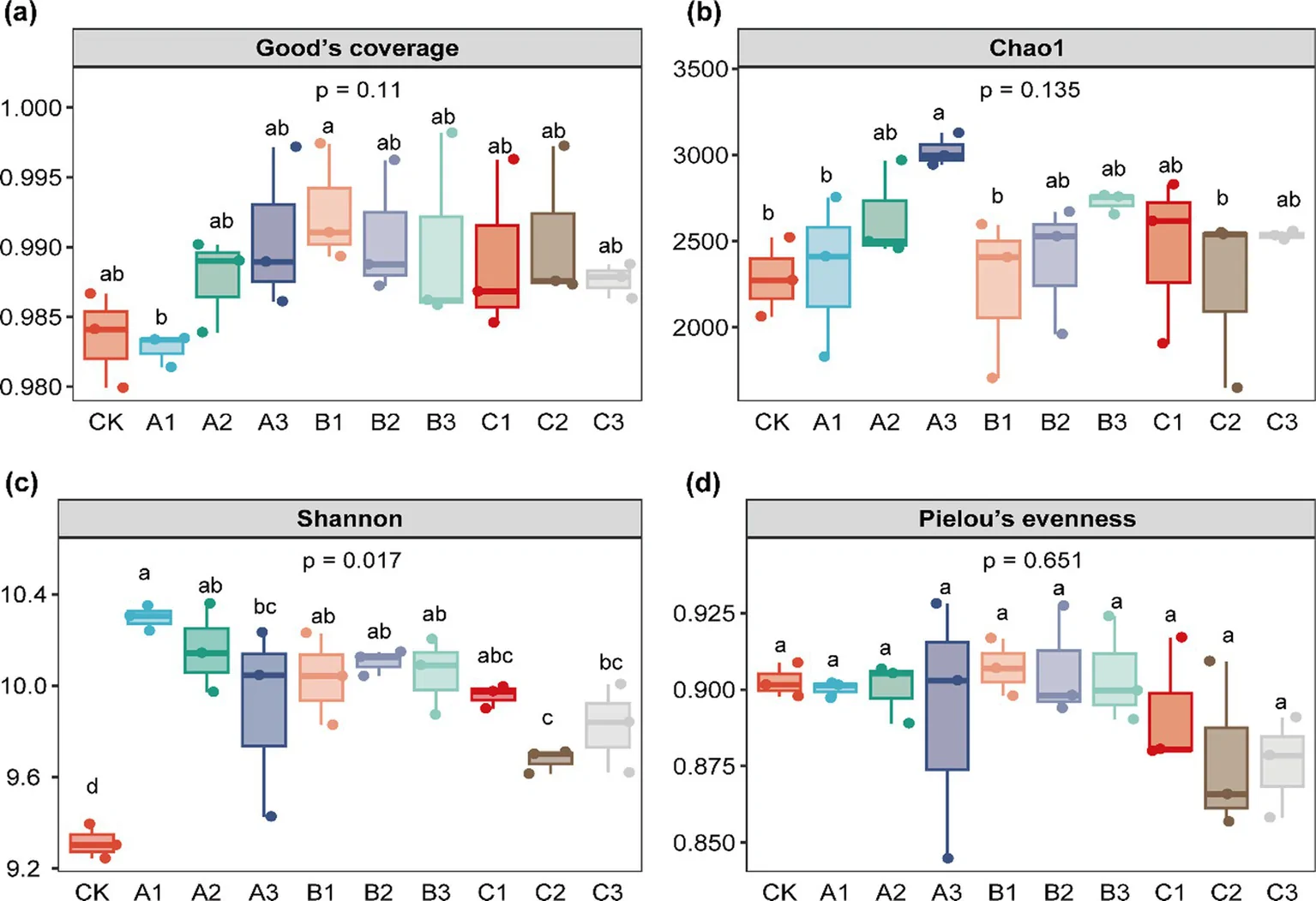
Effect of amendments on bacterial alpha diversity. **(a)** Good’s coverage, the sample coverage index. **(b)** Chao1, the species richness index. **(c)** Shannon, the diversity index. **(d)** Pielou’s evenness, the sample evenness index. Error bars represent the standard deviation of triplicates. Data are presented as mean ± standard error (*n* = 3). The same lowercase letters mean no significant difference, while different lowercase letters mean significant differences between groups (ANOVA, LSD test, *p* < 0.05).

The rarefaction curves and abundance curves of all observed ASVs indicated that the sequencing depth acquired from this research was enough to capture most of the bacterial information and confirmed that the quantities of sequences were adequate to reveal the diversity of the bacterial community (Supplementary Figure S3). Alpha diversity indices were used to evaluate the diversity and abundance of soil microorganisms. The Good’s coverage of bacterial ASVs was all above 98.0%, which suggested that community sampling was almost complete (Figure 8a). Figures 8b,c indicate that all treatments increased the Chao1 and Shannon indices. Compared to CK, A3 significantly enhanced Chao1 indices by 32.27% (*p* < 0.05), and all treatments significantly increased Shannon indices by a range from 3.89% (C2) to 10.58% (A1) (*p* < 0.05). Biochar-based amendments could significantly enhance the soil microbial diversity indices (*p* < 0.05) but did not significantly increase richness indices (*p* < 0.05). The Pielou_e indices (Figure 8d) for all treatments were more than 85%, suggesting that the samples were homogeneous in bacterial community composition.

According to the PCoA plot, there was obvious dispersion among the treatments (Supplementary Figure S4), which suggested that there were remarkable variations in β-diversity among them, especially by comparison with CK; however, A1 was more similar to CK. Furthermore, PCoA1 contributed 12% of the variation among treatments, whereas PCoA2 contributed 8.9%. It indicated that the biochar-based amendments contributed to the variation in the bacterial community.

### 3.6 Structure and composition of soil bacterial communities

**Figure 9:**
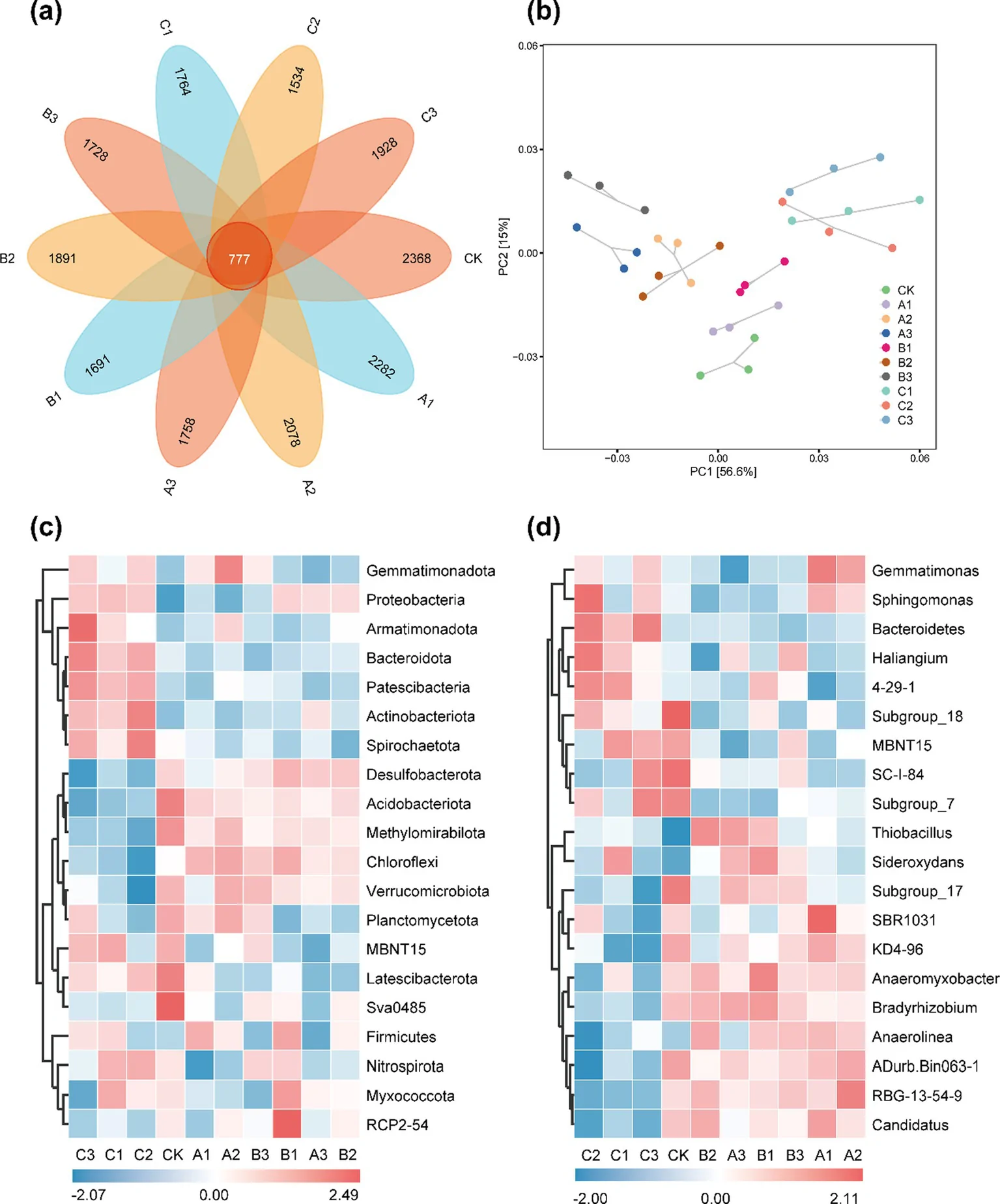
**(a)** Venn diagram representation of the ASVs shared among the different treatments. **(b)** Application of Principal Component Analysis (PCA) to classify the contribution and distribution of microbial compositional similarities. **(c,d)** The clustering heatmap of the bacterial community at the phylum and genus level during biochar-amended soil.

As illustrated in Figure 9a, 3,145, 3,095, 2,855, 2,535, 2,468, 2,668, 2,505, 2,541, 2,311, and 2,705 ASVs were identified in CK, A1, A2, A3, B1, B2, B3, C1, C2, and C3 individually. As indicated in the Venn diagram, there were a total of 19,799 ASVs detected in one or more of the 10 treatments, which represented the core bacterial ASVs. Correspondingly, there were 2,368, 2,282, 2,078, 1,758, 1,691, 1,891, 1,728, 1,764, 1,534, and 1,928 ASVs identified by exactly one treatment, which comprised the group-specific particular ASVs.

PCA revealed that the structure of the bacterial communities altered markedly with the different treatments (Figure 9b). PC1 and PC2 accounted for 56.6 and 15% of the variation in bacterial communities between treatments, individually. Similar to Figure 9c, Supplementary Figure S5 was a map of species taxonomic loadings, illustrating that *Thiobacillus*, *Anaerolinea*, *RBG-13-54-9* (*p_Chloroflexi*), *Subgroup_6* (*p_Acidobacteria*), and *SBR1031*(*p_Chloroflexi*) made a large contribution to the grouping variance.

The top 30 bacterial abundances at the phylum level were presented in Figure 9c. In detail, the predominant bacteria in CK were *Desulfobacterota*, *Acidobacteriota*, *Methylomirabilota*, *Verrucomicrobiota*, *Planctomycetota*, *MBNT15* (*p_Proteobacteria*), *Latescibaterota*, and *Sva0485* (*p_Proteobacteria*). A1, A2, A3, B1, B2, and B3 identified that the most abundant bacteria were *Desulfobacterota*, *Acidobacteriota*, *Methylomirabilota*, *Chloroflexi*, and *Verrucomicrobiota*. Nevertheless, C1, C2, and C3, an enriched bacterial community was exhibited in *Gemmatimonadota*, *Proteobacteria*, *Armatimonadota*, *Bacteroidota*, *Patescibacteriota*, and *Spirochaetota*. At the level of genus (Figure 9d), the predominant bacteria in CK were *Subgroup_18* (*p_Acidobacteria*), *SC-I-84* (*p_Proteobacteria*), *Subgroup_7* (*p_Acidobacteria*), *Subgroup_17* (*p_Acidobacteria*), *ADurb*. *Bin063-1* (*p_Verrucomicrobia*), and *MBNT15* (*p_Proteobacteria*). A1, A2, A3, B1, B2, and B3 identified that the most abundant bacteria were *Anaeromyxobacter*, *Bradyrhizobium*, *Anaerolinea*, *Candidatus_Solibacter*, *RBG-13-54-9* (*p_Chloroflexi*), and *ADurb. Bin063-1* (*p_Verrucomicrobia*). C1, C2, and C3, an enriched bacterial community was exhibited in *Bacteroidetes_vadinHA17*, *Haliangium*, *4–29-1* (*p_Nitrospirae*), *Sphingomonas*, and *MBNT15* (*p_Proteobacteria*).

### 3.7 Biomarkers and the co-occurrence network of the bacterial community

**Figure 10:**
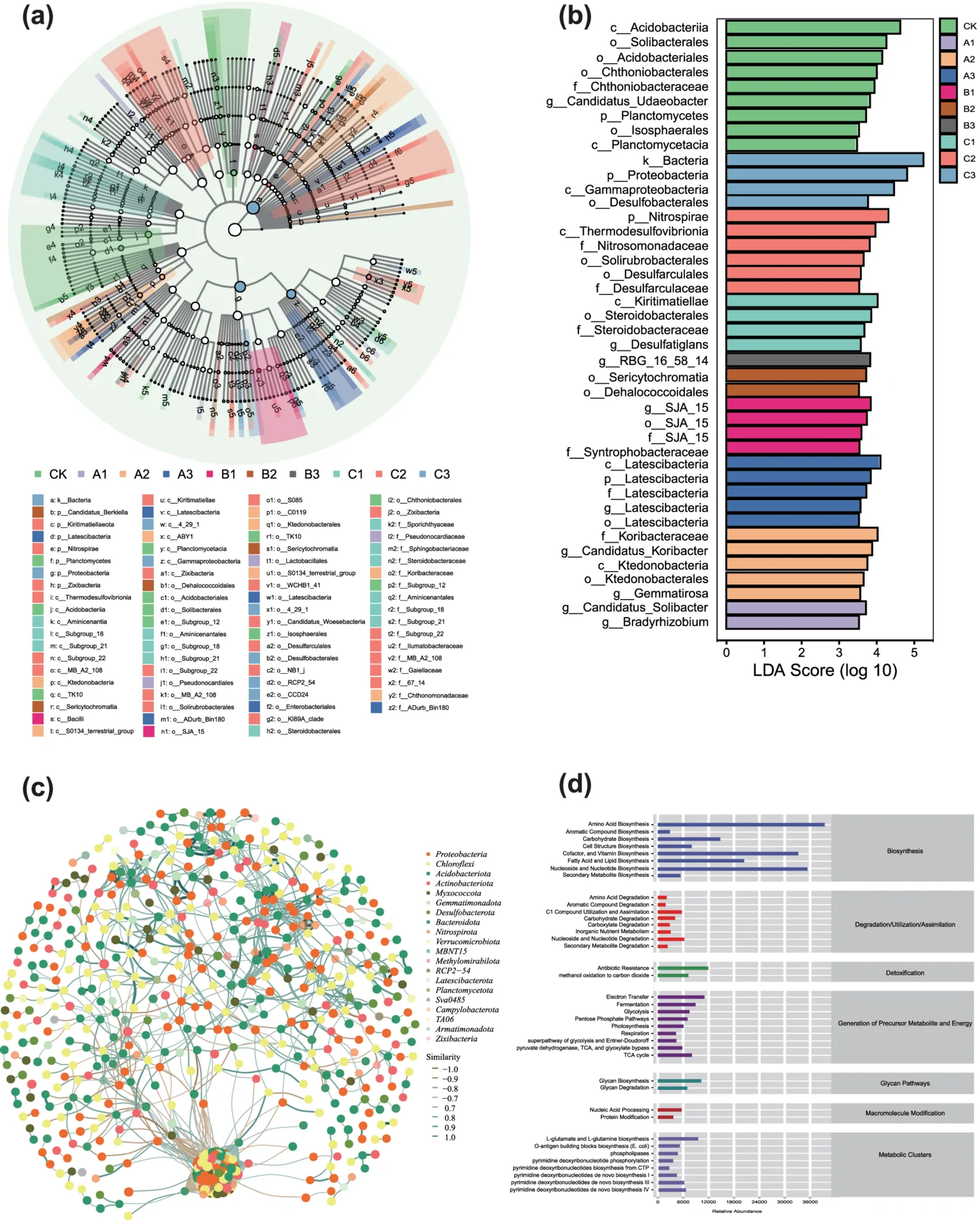
**(a)** LefSe taxonomic cladogram. The colored nodes from the inner circle to the outer circle represented the hierarchical relationship of all taxa from the phylum to the genus level. The diameter of each small circle represented the taxa’s abundance. **(b)** LDA score. Enriched taxa with an LDA score > 3.6 were shown in the histogram. The greater the LDA score was, the more significant the phylotype microbiota was in the comparison. **(c)** Bacterial Network, at the phylum level, with the top 20 and abundance 800 communities. **(d)** The community gene function prediction of soil with the addition of biochar-based amendments.

To identify the main microflora by taxonomy, evolutionary cluster analyses were performed in Figure 10a. As illustrated in the cladogram, *Acidobacteriia*, *TK10* (*p_Chloroflexi*), and *Planctomycetacia* had the most abundant flora in the green areas (CK). *Pseudonocardiales*, *Lactobacillales*, and *Pseudonocardiaceae* had the highest flora in A1 treatment, *Ktedonobacteria* and *S0134*_terrestrial_group (*p_Gemmatimonadetes*) in A2, *Latescibacteria* in A3, *Bacilli* in B1, *Candidatus_Berkiella* and *Sericytochromatia* in B2, *Ktedonobacterales* in B3, *Aminicenatia*, *Subgroup_18* (*p_Acidobacteria*), *Subgroup_21* (*p_Acidobacteria*) in C1, *Kiritimatiellaeota*, *Nitrosirae*, *Zixibacteeria*, and *Thermodesulfovibrionia* in C2, *Proteobacteria* and *Gammaproteobacteria* in C3. In conclusion, these results suggested that biochar-based amendments altered the key biomarkers of soil microbiota in rice fields and promoted the reproduction of specific bacteria.

To determine the main biomarkers of soil microbiota in different groups, we conducted LefSe analysis between all treatments. LDA (Figure 10b) revealed nine distinguishing characteristics in CK (LDA > 3.5, *p* < 0.05), *Acidobacteriia*, *Solibacterales*, *Acidobacteriales*, and *Chthoniobacteraceae* were the dominant microbiota. The predominant microbiota (LDA > 3.5, *p* < 0.05) of biochar-based amendments were *Candidatus_Solibacter*, and *Bradyrhizobium* in A1, *Ktedonobacterales* and *Candidatus_Koribacter* in A2, *Latescibacteris* in A3, *SJA_15* (*p_Chloroflexi*) in B1, *Sericytochromatia* and *Dehalococcoidales* in B2, *RBG_16_58_14* (*p_Chloroflexi*) in B3, *Kiritimatiellae* and *Steroidobacterales* in C1, *Nitrospirae*, *Thermodesulfovibrionia*, and *Nitrosomonadaceae* in C2, *Bacteria*, *Proteobacteria*, *Gammaproteobacteria*, and *Desulfobacterota* in C3.

The correlations among microorganisms of biochar-based amendments applied for cadmium management in agricultural soil were assessed by co-occurrence networks. The co-occurrence networks of the top 20 microorganisms with an abundance of 800 at the phylum level were illustrated in Figure 10c. All biochar-based treatments had higher average degrees than CK (Table 6), and B2, B3, C2, and C3 were significantly higher than CK (*p* < 0.05). The average length and closeness centrality of all treatments differed little. The network of the CK had 1,703 nodes and 15,935 edges (Table 6). Compared to CK, all treatments decreased the nodes, and most treatments decreased the edges, with A1, B3, C2, and C3 treatments exhibiting a significant decrease in the number of nodes and edges (*p* < 0.05). Similarly, modularity was significantly reduced in all biochar treatments (*p* < 0.05). In summary, in combination with the described microbial abundance characteristics, the addition of biochar amendments regulated the microbial community succession process and had an antagonistic influence on the sophisticated soil bacterial community network.

**Table 6:** Co-occurrence topological features of bacterial networks in each treatment.

Treatment	Average degree	Average length	Closeness centrality	Density	Average cluster	Nodes	Edges	Modularity
CK	25.27 ± 0.91e	5.90 ± 0.06ab	1.29 ± 0.11ab	0.0110 ± 0.0008e	36.67 ± 4.16bc	1,705.33 ± 77.42a	15,935.67 ± 204.08a	0.33 ± 0.01a
A1	27.25 ± 0.84cde	5.93 ± 0.14ab	1.12 ± 0.08bc	0.0141 ± 0.0013 cd	44.00 ± 5.29ab	1,469.67 ± 71.14b	15,146.67 ± 57.84b	0.29 ± 0.01b
A2	26.45 ± 1.11cde	5.91 ± 0.13ab	1.29 ± 0.17ab	0.0128 ± 0.0014cde	36.33 ± 5.03bc	1,562.00 ± 83.37ab	15,514.00 ± 190.90ab	0.31 ± 0.01ab
A3	25.87 ± 2.18de	5.76 ± 0.02b	1.38 ± 0.06a	0.0116 ± 0.0026de	32.67 ± 2.52c	1,678.67 ± 205.37a	15,958.33 ± 715.21a	0.33 ± 0.02a
B1	26.26 ± 1.24de	6.00 ± 0.12a	1.26 ± 0.11ab	0.0123 ± 0.0012cde	37.00 ± 5.57bc	1,598.33 ± 103.25ab	15,611.00 ± 424.49ab	0.30 ± 0.02b
B2	27.71 ± 0.97bcd	5.82 ± 0.02ab	1.26 ± 0.05ab	0.0129 ± 0.0012cde	38.33 ± 0.58abc	1,578.33 ± 77.67ab	15,999.00 ± 82.05a	0.31 ± 0.01ab
B3	28.60 ± 0.44bc	5.81 ± 0.05ab	1.30 ± 0.21ab	0.0148 ± 0.0007c	36.00 ± 7.00bc	1,440.00 ± 48.12b	15,300.67 ± 300.29b	0.29 ± 0.02b
C1	27.27 ± 1.00cde	5.76 ± 0.06b	1.16 ± 0.15abc	0.0134 ± 0.0016cde	43.00 ± 7.55ab	1,526.00 ± 111.85ab	15,435.67 ± 328.52ab	0.30 ± 0.01b
C2	29.53 ± 1.05ab	5.89 ± 0.16ab	1.10 ± 0.03bc	0.0203 ± 0.0011b	38.00 ± 3.46abc	1,140.67 ± 33.86c	13,188.67 ± 204.68c	0.20 ± 0.01c
C3	31.41 ± 1.27a	5.87 ± 0.23ab	0.99 ± 0.04c	0.0229 ± 0.0021a	46.67 ± 2.08a	1,064.00 ± 51.00c	12,898.67 ± 87.13c	0.19 ± 0.01d

Data are presented as mean ± standard error (*n* = 3). Same lowercase letters mean no significant difference while different lowercase letters mean significant differences between groups (ANOVA, LSD test, *p* < 0.05).

The function of high-quality bacterial genes in the population was analyzed by PICRUSt, as shown in Figure 10d. The addition of biochar impacted the function of amino acid, aromatic compound, carbohydrate, cofactor, vitamin, fatty acid, lipid, nucleotide, and secondary metabolite biosynthesis and degradation, C1 compound utilization and assimilation, inorganic nutrient metabolism, electron transfer, glycolysis, photosynthesis, TCA cycle, and glycan biosynthesis and degradation.

### 3.8 The correlations between soil physiochemical properties, DTPA-cd, bacterial communities, and rice yield

**Figure 11:**
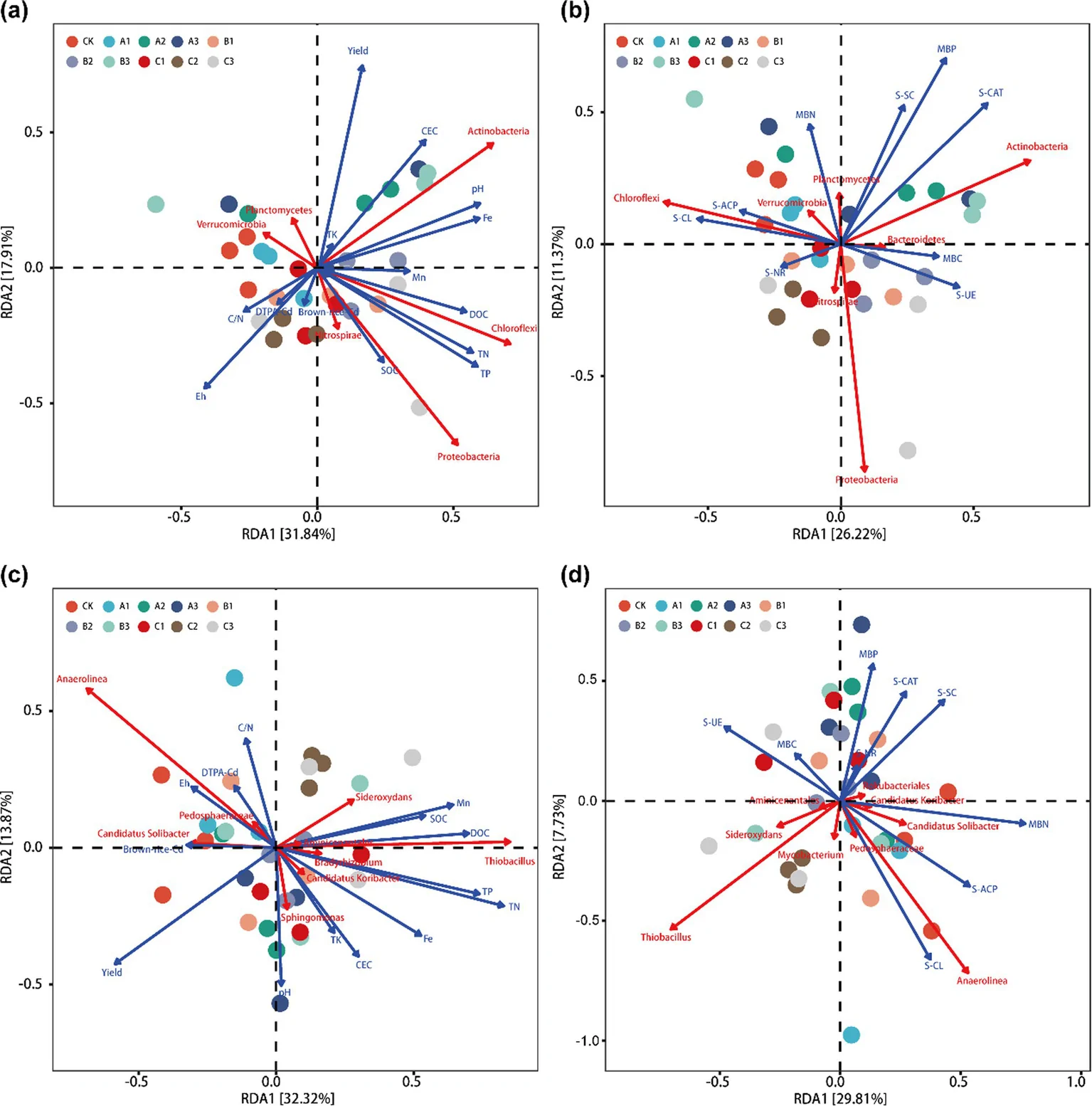
**(a)** Redundancy analysis (RDA) of soil physicochemical properties on bacterial community at the phylum level. **(b)** RDA of soil enzyme activities and bacterial communities at the phylum level. **(c)** RDA of soil physicochemical properties on bacterial community at the genus level. **(d)** RDA of soil enzyme activities and bacterial communities at the genus level.

Redundancy analysis (RDA) was conducted to better evaluate the interactions among soil physiochemical properties, DTPA-Cd, rice yield, enzyme activity, soil microbial biomass, and bacterial communities at the phylum and genus levels in Figure 11. The closer the distance between these points, the smaller the difference between the microbial communities of these samples. The arrows represented the degree of correlation between variations, and the longer the arrow, the greater the correlation. Furthermore, the angle between arrows indicated the correlation, with an acute angle suggesting a positive correlation and an obtuse angle implying an anticorrelation. According to Figure 11a, the first axis of environmental factors accounted for 31.84%, and the second axis for 17.91% of the observed variation. The bacterial community structure at the phylum level was mainly influenced by pH, Eh, TP, and TN, whereas DTPA-Cd was mostly affected by *Chloroflexi*, *Proteobacteria*, and *Actinobacteria*. As shown in Figure 11b, two axes of the RDA explained 37.59% of enzyme activity and soil microbial biomass variation in bacterial community (RDA1 = 26.22%, RDA2 = 11.37%). S-CL, S-ACP, and S-NR were positively correlated with *Chloroflexi* and *Verrucomicrobia*, and negatively correlated with *Actinobacteria* and *Bacteroidetes*. However, S-SC, S-CAT, and S-UE were in contrast. MBN, MBP, and MBC were positively correlated with *Verrucomicrobia*, *Planctomycetes*, *Actinobacteria*, and *Bacteroidetes*, and negatively correlated with *Proteobacteria* and *Nitrospiae*. As described in Figure 11c, 32.32% was explained by RDA1, 13.87% by RDA2. DTPA-Cd was positively correlated with the bacterial community structure at the genus level of *Anaerolinea*, *Pedosphaeraceae*, and *Candidatus Solibacter*, and negatively correlated with *Sphingomonas*, *Bradyrhizohium*, and *Candidatus Koribactor*. As illustrated in Figure 11d, two axes of the RDA explained 37.54% (RDA1 = 29.81%, RDA2 = 7.73%). *Anaerolinea*, *Thiobacillus*, *Candidatus Solibacter*, *Pedosphaeraceae*, and *Mycobacterium* were positively correlated with S-CL, S-ACP, and MBN, whereas negatively correlated with S-UE, MBC, and MBP.

**Figure 12:**
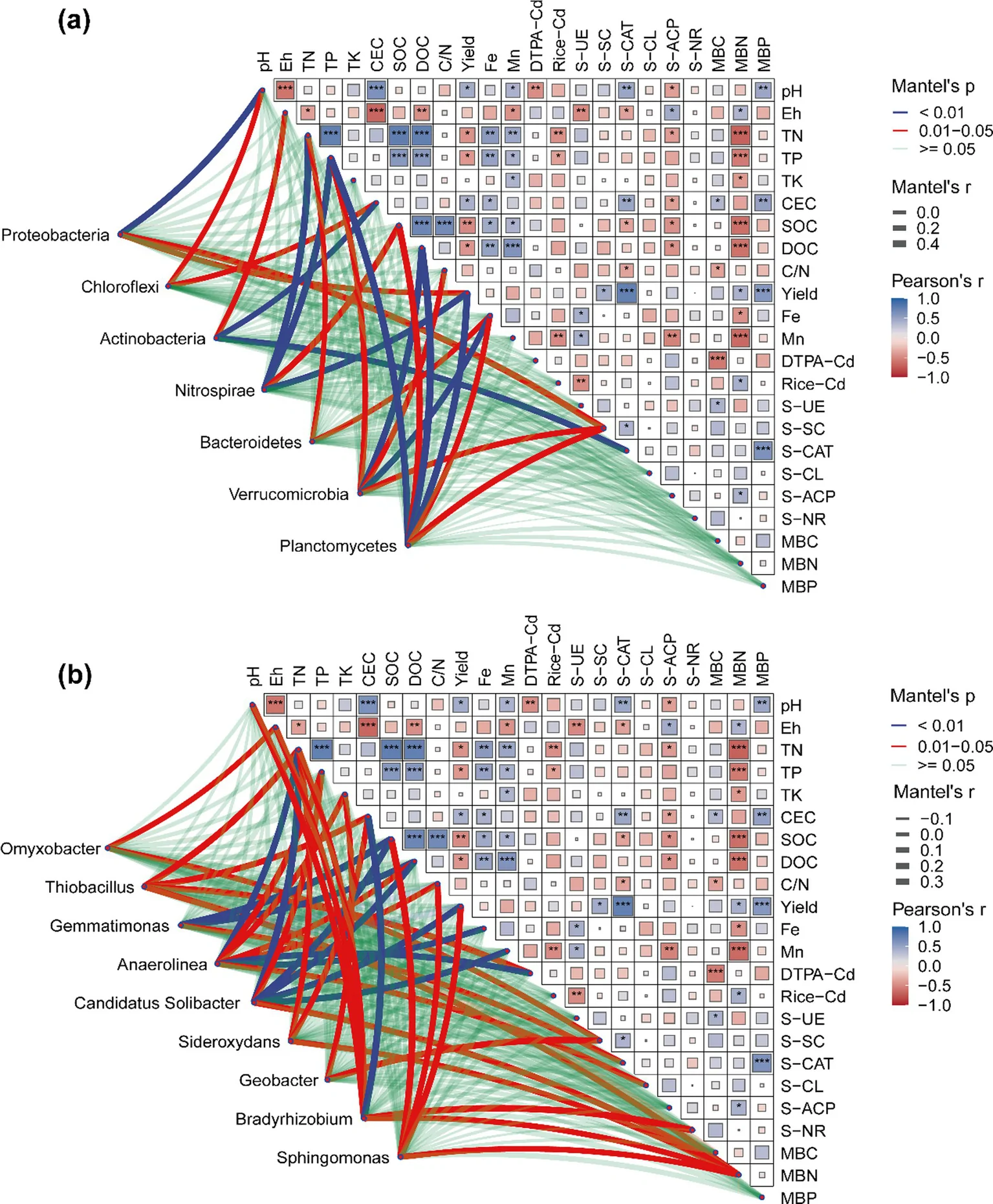
Relationships between soil physicochemical properties, enzyme activity, microbial biomass, and microorganisms at the phylum **(a)** and genus **(b)** levels. Line color responds to the level of significance. Line width indicates Mantel’s *r* value. Pearson’s *r* is the correlation coefficient between soil physicochemical properties, enzyme activity, and microbial biomass, indicated by the scale of the individual grids and the shade of the colors.

In order to confirm the independent influence of environmental factors on different bacteria, we performed a mental test to correlate the taxonomic combinations at the 7 phylum level and 9 genus level of the highest abundance with the environmental factors (Figure 12). The phylum (*Proteobacteria*, *Chloroflexi*, *Actinobacteria*, *Nitrospirae*, *Bacteroidetes*, *Verrucomicrobia*, and *Planctomycetes*) had a significant association with pH, TN, TP, CEC, SOC, DOC, Fe, S-SC, and S-CAT (Figure 12a). The genus (*Anaeromyxobacter*, *Thiobacillus*, *Gemmatimonas*, *Anaerolinea*, *Candidatus Solibacter*, *Sideroxydans*, *Bradyrhizobium*, and *Sphingomonas*) had a significant association with the majority of soil physicochemical properties, DTPA-Cd, rice yield, enzyme activity, and microbial biomass (Figure 12b).

## 4 Discussions

### 4.1 Effects on soil physiochemical properties, DTPA-cd, soil enzyme activity, and soil microbial biomass

The elemental stoichiometry and spatial-distribution patterns of amendments serve as key intrinsic factors determining their physicochemical properties and inherent Cd-immobilization potential. The three biochar samples exhibited high carbon proportions with low H/C and O/C molar ratios, indicating sufficient pyrolytic carbonization and well-developed aromatic frameworks (Zaini et al., 2023). Such aromatic carbon skeletons could supply abundant sites for heavy-metal sequestration via *π*–*π* interactions (Tong et al., 2019). The subtle spatial heterogeneity of carbon signals observed from EDS-mapping originated from the intrinsic lignocellulosic microstructures of feedstock biomass. In contrast, distinctly higher H/C and O/C molar ratios for ROF and MFA reflected abundant surface-active O/N-containing moieties (–COOH, –OH, –NH₂), which enabled effective Cd retention through complexation and surface proton-exchange reactions. ROF contained relatively high contents of S, sulfur-derived thiol groups endowed materials with favorable heavy-metal chelating performance, whereas mineral-bound K and Mg participated in cation-exchange processes. As an inorganic phosphate-dominated material, CMPF possessed negligible organic components, but abundant Ca, Mg, and P. Homogeneously dispersed Ca–Mg–P mineral phases thermodynamically facilitated the formation of cadmium-phosphate precipitates, representing a critical geochemical pathway for Cd passivation (Guo et al., 2023). Obvious discrepancies in mineral-element fingerprints among different amendments revealed strong feedstock dependence. Bulk elemental analysis results were corroborated by EDS elemental-mapping, which directly visualized the spatial differentiation between organic carbon skeletons and inorganic mineral fractions.

Biochar-based composite amendments comprehensively improved soil physicochemical indicators via synergistic multiple pathways. In our research, applying biochar-based amendments significantly increased soil pH, which was consistent with previous studies (Chen et al., 2022; Khaliq et al., 2024; Xu et al., 2022). For soil acidity neutralization, high-ash alkaline minerals and exchangeable Ca^2+^, Mg^2+^, K^+^ cations inherent in biochar, combined with abundant alkaline cations released from CMPF, continuously neutralize free H^+^ in soil; meanwhile, negatively charged functional groups (–OH, –COOH, –SH) on biochar surfaces immobilize hydrogen ions, jointly elevating soil pH, and CMPF contributes more prominently to acid buffering (Bagheri Novair et al., 2023; Zhang et al., 2023). Increased pH generates abundant hydroxyl anions and more active adsorption sites on soil colloids, which immobilize Cd through electrostatic attraction and hydroxide coprecipitation and restrain Cd uptake by rice (Hong et al., 2022; Wei et al., 2023); Additionally, pH alteration indirectly modulates soil enzyme activities and microbial community composition (Alaboudi et al., 2019). In terms of redox regulation, composite amendments show a significant negative correlation with soil Eh. Abundant reductive functional groups on biochar consume oxidants, and the porous framework provides microhabitats for microbial colonization, which further deplete soil oxygen and create a moderately reducing microenvironment (Yang et al., 2021). Moderately low Eh transforms labile Cd into insoluble sulfide and carbonate fractions and limits crop Cd accumulation, yet excessive reduction should be avoided to prevent toxic hydrogen sulfide generation (Luo et al., 2020; Xu et al., 2023). Regarding nutrient retention and Cd immobilization, amendments with higher biochar proportions achieve the greatest CEC improvement. The porous structure of biochar expands soil specific surface area and enhances ion exchange capacity via oxygen-containing functional groups; simultaneously, soluble cations from CMPF, dissociated carboxyl/phenolic hydroxyl groups, and chelating sites of MFA collectively boost soil cation adsorption capacity (Hailegnaw et al., 2019; Yang et al., 2021). Elevated CEC immobilizes Cd on soil colloids through cation exchange and lowers DTPA-Cd concentrations (Hussain Lahori et al., 2017), while also improving soil aggregate structure and water retention capacity to enhance field water-fertilizer preservation and anti-erosion performance (Arabi et al., 2018). Collectively, the composite system integrating biochar, CMPF, and MFA achieves integrated regulation of soil acidity, redox status, and cation retention, overcoming the limitation of pure biochar that merely supplies carbon without sufficient acid buffering and phosphorus input. Such multi-functional amendments exhibit superior physicochemical improvement potential for mildly Cd-polluted acidic paddy soils, while further field optimization of mixing ratios and application rates is required across soils with divergent baseline pH and clay contents.

Our research demonstrated that biochar-based amendments significantly decreased the DTPA-Cd concentration. This result was in accordance with previous works (Qi et al., 2023; Li J. et al., 2022; Li X. et al., 2022; Liu et al., 2022; Xiong et al., 2019; Chen et al., 2021). The immobilization of Cd by biochar-based composite amendments relies on the synergistic effect of multiple reactions, including physical adsorption, ion exchange, complexation, and co-precipitation (Chen et al., 2021; Wu et al., 2020). The well-developed porous architecture of biochar supplies abundant physical adsorption sites, and a higher specific surface area corresponds to a stronger Cd retention capacity (Nguyen et al., 2023). Oxygen-containing functional groups (–OH, –COOH, C=O) on biochar surfaces bind Cd^2+^ via ion exchange and coordination complexation; alkaline metal cations (Ca, Mg, K, Na) also exchange with Cd ions, while elevated soil CEC increases negative surface charge to enhance electrostatic adsorption (Alaboudi et al., 2019; Lv et al., 2021). As a key component, CMPF passivates Cd through two pathways: it raises soil pH to transform labile Cd into insoluble hydroxide and carbonate precipitates, reducing exchangeable Cd and increasing Fe–Mn oxide-bound Cd (Liang et al., 2022; Wei et al., 2023); abundant Ca^2+^ and Mg^2+^ compete with Cd^2+^ for colloidal adsorption sites to cut crop Cd uptake (Zanin Lima et al., 2023; Zhang et al., 2023). Native soil CO₃^2−^ and PO₄^3−^ additionally precipitate Cd ions (Lahori et al., 2017). Organic matter from ROF and MFA forms stable organometallic chelates with Cd^2+^ through functional groups and stimulates soil microorganisms to mediate Cd immobilization. MFA further generates Fe/Al-containing adsorptive colloids and optimizes the surface adsorption properties of soil particles. Collectively, these coupled pathways reduce Cd mobility and bioavailability in paddy soils (Hong et al., 2022; Wang et al., 2024; Xiang et al., 2022; Zuo et al., 2023).

Soil enzyme activities serve critical roles in diverse physiochemical processes and act as vital bioindicators for evaluating soil health. S-UE catalyzes the hydrolysis of urea to ammonia or ammonium ions, which mirrors the nitrogen supply capacity of paddy soils (Sun et al., 2013). In our study, biochar-based amendments significantly elevated soil S-UE activity, which was beneficial for nutrient uptake. Wen et al. (2021) demonstrated that elevated soil pH could stimulate S-UE activities; moreover, soil S-UE was secreted in abundance when the soil pH was 5.5–6.0. S-SC is a key indicator reflecting soil maturity and fertility, and it exerts pivotal functions in supporting crop growth (Stpniewska et al., 2009). Biochar-based composite amendments generated non-significant variations in S-SC and S-CL activities, which may be attributed to biochar application dosage, field application patterns, and inherent intrinsic soil properties. S-NR acts as a core critical enzyme in the soil nitrate assimilation pathway. Excessively elevated S-NR activity accelerates nitrogen loss and drives denitrification to produce hydroxylamine, which can be further reduced to ammonium and ultimately disrupt soil nitrogen balance (Reda et al., 2011; Watanabe, 2009). Our results revealed that biochar-based composite amendments increased S-NR activity overall, while this activity declined as the proportion of biochar within the amendments rose. Such a trend is likely associated with the proportion of ROF in the composite materials: higher organic fertilizer contents supply more available nutrients, which in turn stimulate S-NR activity. S-ACP serves as a vital driver for soil organic phosphorus mineralization. It catalyzes the hydrolysis of phosphate monoesters to release inorganic phosphate, thereby participating in biological phosphorus metabolism (Bhattacharyya et al., 2008). Our results revealed a significantly negative correlation between S-ACP activity and the application proportion of biochar in composite amendments. Consistent with our findings, Minling et al. (2021), reported that corn straw-derived biochar and Fe–Mn oxide-modified biochar suppressed S-ACP activity. Similarly, Chen et al. (2020) also documented the inhibitory effect of biochar on this enzyme. Such reductions in S-ACP activity can be primarily attributed to elevated soil pH, which is further validated by the correlation analysis in Figure 12, showing a significant negative correlation between pH and S-ACP activity (*p* < 0.05). Frankenberger and Johanson (1982) demonstrated that the preferred pH for S-ACP activity is 4.0–5.0. Since biochar-based composite amendments markedly raised soil pH beyond this suitable range, S-ACP activity was consequently inhibited. S-CAT is a redox enzyme that accelerates the degradation of hydrogen peroxide and protects microorganisms from peroxide toxic stress, which is widely adopted as a core bioindicator to evaluate soil fertility, reflecting the intensity of soil humification and organic matter transformation rates. Our results indicated that S-CAT activity slightly increased with the rising proportion of biochar-based composite amendments, yet this increment was statistically insignificant. Moreover, S-CAT activity exhibited significant positive correlations with soil pH and CEC, and a remarkable negative correlation with soil Eh. It might be that maize biochar and bamboo biochar application improved soil aeration and water retention, as well as increased soil organic matter and dissolved organic matter content, thus increasing soil microbial activity and microbial metabolism (Sun et al., 2016).

Soil microbial biomass represents a labile, critical fraction of soil organic matter that governs nutrient supply turnover and is therefore universally recognized as a reliable indicator of soil fertility. Its dynamics are strongly modulated by different soil amendment regimes (Singh et al., 2016). In our research, as illustrated in Figure 6, biochar-based amendments significantly increased MBC, MBN, and MBP compared to CK (*p* < 0.05), which aligns with multiple previous field and incubation observations (Singh et al., 2018; Wang et al., 2021; Zhu et al., 2017). Such pronounced accumulation of microbial biomass can be attributed to the optimized soil microenvironment driven by amendments, which facilitates microbial colonization and enrichment (Steiner et al., 2008). Most recently, Duan et al. (2023a,b), Chen et al. (2021), Yuan et al. (2023), and Zhang et al. (2023) further confirmed that persistent biochar application reshapes the composition and potential metabolic functions of soil bacterial communities. Warnock et al. (2010) proposed that the intricate porous architecture of biochar could serve as shelters or microspheres for microorganisms to colonize, where they could survive and propagate well. Meanwhile, the dark surface of biochar enhances heat absorption capacity, which satisfies the thermal demand for rapid microbial propagation. Additionally, soil microbial biomass and bacterial gene abundance were increased by improved soil chemical properties and the high ash, nitrogen, and phosphorus content of biochar-based amendments (Azeem et al., 2023). Therefore, in this research, biochar-based amendments treated soil-enriched microbial communities and improved soil physicochemical properties, thereby increasing the microbial biomass of soil.

### 4.2 Responses of the bacterial community to biochar-based amendments

Microbial diversity is critical in maintaining the productivity, sustainability, and recoverability of ecosystems, as microorganisms perform an essential role in modulating organic matter biodegradation and nutrient mobilization. Their diversity and community structure are jointly influenced by plant populations, soil characteristics, and climatic conditions (Yuan et al., 2023; Zhang et al., 2018). The fitness of bacteria to environmental variations may be evaluated by their diversity and functional properties (Xiao et al., 2022). Application of biochar-based amendments to rice fields reshapes soil properties and alters soil microbial activities both directly and indirectly (Gao et al., 2021). Specifically, Chao 1 indices increased with higher biochar content, suggesting enhanced bacterial community richness in the amendment treatments. Meanwhile, Shannon indices of all treatments were significantly higher than CK, demonstrating that biochar-based amendments effectively increased soil microbial diversity, which is consistent with previous studies (Li J. et al., 2022; Li X. et al., 2022; You et al., 2021; Zhang et al., 2019). Mechanistically, biochar-based amendments provided direct nutrients for soil bacterial growth (Duan et al., 2023a); moreover, they significantly increased soil pH, TN, TP, CEC, SOC, and DOC, remodeling the soil microenvironment to promote higher bacterial abundance and diversity (Deshoux et al., 2023; Luis Moreno et al., 2022). Furthermore, the large specific surface area and mesoporous structure of biochar could absorb inorganic ions and soluble organic matter, providing favorable microhabitats for bacterial survival and colonization (Chen et al., 2018; Chintala et al., 2014; Zhou et al., 2017).

In our research, *Proteobacteria*, *Chloroflexi*, *Acidobacteriota*, and *Actinobacteriota* were identified as the dominant bacterial phyla across all treatments. *Proteobacteria* generally account for the largest proportion of bacteria in soil ecosystems, as they preferentially utilize readily degradable organic materials in soil; their abundance decreases when such labile substrates are depleted (Ran et al., 2023; Wang et al., 2021). *Lysimycetes* affiliated with *Proteobacteria* exhibit prominent antagonistic effects on various plant pathogenic fungi and possess salt tolerance (Hayward et al., 2010). At the phylum level, biochar-based amendments insignificantly and slightly increased the relative abundance of *Proteobacteria*, which may be attributed to the nutrient-rich properties of the amendments. *Chloroflexi* is more abundant in nutrient-rich environments and preferentially utilizes unstable carbon sources, facilitating soil organic matter degradation (Narsing Rao et al., 2022). Our study observed that maize and bamboo biochar-based amendments slightly increased the abundance of *Chloroflexi*, while coconut shell activated carbon-based amendments slightly decreased it. We also discovered that all treatments reduced the abundance of *Acidobacteriota*, consistent with Zhang et al. (2023) and Luis Moreno et al. (2022). *Acidobacteriota* were typically oligotrophic and thrived in infertile soil (Zhang et al., 2014), so their decreased abundance is likely due to soil eutrophication induced by biochar-based amendments. Distinctively, Kim et al. (2021) suggested that high pH and Zn metal might increase *Acidobacteriota* abundance, while Cu, Cd, As, Cr, SOM, and moisture content had no influence on *Acidobacteriota* communities. Gao et al. Chen et al. (2021) found that biochar application increased *Acidobacteriota* abundance, which might be related to its disease suppression. *Actinobacteriota* was another highly abundant soil bacterial phylum, whose abundance increases under moisture-restricted conditions or in biochar-amended soils (Zheng et al., 2018). Yuan et al. (2023) showed that the combined application of nitrogen fertilizer and biochar significantly increased *Actinobacteriota* abundance, and Bello et al. (2020) emphasized its crucial roles in degrading complex compounds, inhibiting pathogenic microorganisms via antibiotic secretion, and solubilizing phosphate. At the genus level, biochar-based amendments significantly increased *Thiobacillus* abundance, with little effect on other genera. *Thiobacillus* increased root availability of nutrients, specifically micronutrients, by oxygenating sulfur and lowering soil acidity (Booali et al., 2024), and Pourbabaee et al. (2020) confirmed that *Thiobacillus* increased maize plant height, shoot weight, and stem diameter. Hence, the increased *Thiobacillus* abundance contributed to crop growth and development.

The results indicate that applied biochar-based amendments enhanced the architecture, functionality, and stabilization of the soil microbial network. By altering soil physicochemical properties and providing favorable microhabitats and nutrients, these amendments regulate microbial growth, metabolism, and function, mainly involving nutrient partitioning, organic matter degradation, and plant growth promotion.

### 4.3 Interaction among soil physiochemical properties, DTPA-cd, and bacterial communities

Application of biochar-based amendments alters multiple soil physicochemical properties (pH, CEC, Eh, TP, TN, SOC, DOC), which consequently reduced the DTPA-Cd content and restructured bacterial communities, thereby lowering grain Cd and increasing rice yield. RDA identified these environmental variables as key drivers of bacterial assembly, while microbial metabolism reciprocally regulates microbial biomass and enzyme activities, forming bidirectional soil-microbe interactions. Elevated pH induced by amendments alleviates Cd toxicity and reshapes microbial assemblages to facilitate crop production (Ran et al., 2023). Meanwhile, increased SOC and DOC supply fuel microbial metabolism and modulate community composition (Cui et al., 2024). Significant correlations exist between taxa and bioavailable Cd. At the phylum level, nitrospirae positively correlate with DTPA-Cd and grain Cd, whereas *Chloroflexi*, *Proteobacteria,* and *Actinobacteria* show negative associations. At the genus level, *Anaerolinea* and *Pedosphaeraceae* accumulate under high Cd stress, while Cd-resistant functional genera, including *Thiobacillus* and *Sideroxydans* thrive in amended soils. Mantel tests verified that taxa such as *Proteobacteria*, *Planctomycetes*, *Candidatus Solibacter,* and *Sphingomonas* are significantly linked to rice yield. In summary, modified soil physicochemical properties serve as fundamental drivers; they directly immobilize labile Cd and select Cd-tolerant functional microbes via adjusted pH and nutrients. The reconstructed bacterial community further enhances Cd sequestration and nutrient cycling. Such coupled interactions among soil physicochemistry, DTPA-Cd, and bacterial communities underpin the core microecological mechanism for concurrent soil remediation and yield promotion by composite amendments.

### 4.4 Synergistic immobilization mechanisms of biochar-based amendments against DTPA-cd in paddy soils

**Figure 13:**
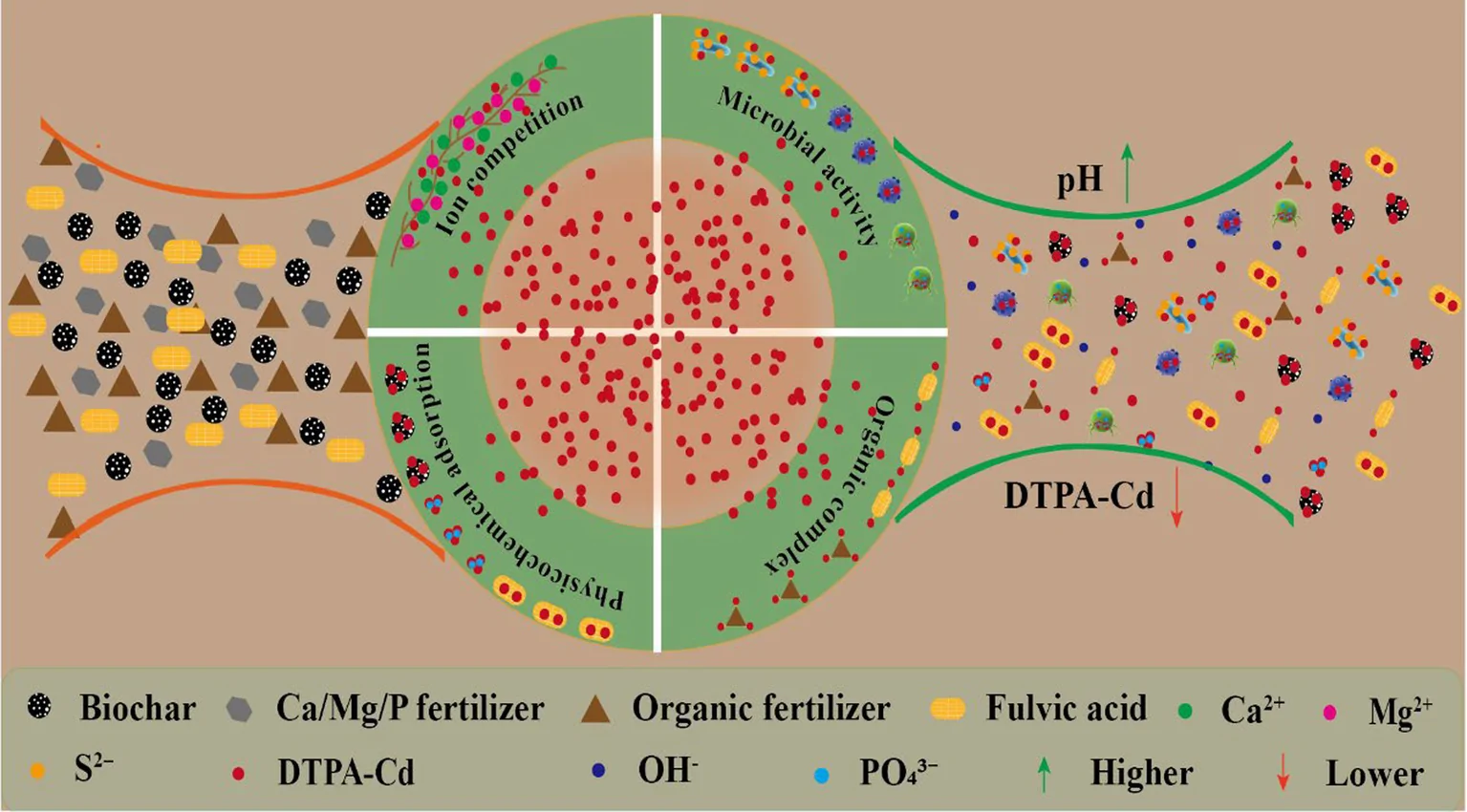
Synergistic mechanism of biochar-based amendment for reducing DTPA-Cd in soil.

The composite synergistic system consisting of biochar, CMPF, ROF, and MFA achieves efficient and long-term cadmium pollution control via multi-stage coupled reactions prioritizing chemical precipitation for primary immobilization, organic complexation for auxiliary stabilization, microbial metabolism for deep passivation, and physical pore structure for long-term sequestration (Figure 13). This integrated strategy not only diminishes soil Cd bioavailability but also simultaneously ameliorates soil fertility and reconstructs healthy soil microecology, bearing great practical significance for heavy metal remediation of farmland and sustainable agricultural development.

(1) Physicochemical synergistic effects: Construction of an “adsorption-precipitation-complexation” barrier. Stage 1, pH regulation and preliminary Cd immobilization. Both biochars and CMPF are alkaline materials that rapidly elevate soil pH and trigger the precipitation of low solubility Cd(OH)₂ and CdCO₃. Under alkaline conditions, phosphate anions released from CMPF react with Cd^2+^ to form insoluble Cd₃(PO₄)₂ and Cd₅(PO₄)₃OH precipitates for further Cd sequestration. Meanwhile, MFA forms negatively charged colloids that encapsulate free Cd^2+^ via electrostatic attraction and block direct Cd exposure to crops. Stage 2, Reinforced organic–inorganic complexation. Abundant oxygen-containing functional groups on organic matter from ROF form stable chelates with Cd^2+^. Additionally, the large specific surface area and porous framework of biochar adsorb free Cd^2+^ in soil solution to suppress its mobility. Cations (Ca^2+^, Mg^2+^) dissociated from CMPF further reduce Cd bioavailability through competitive ion exchange.
(2) Biochemical coupling mechanism: Microbe-driven transformation of cadmium speciation. First, synergistic Cd immobilization via microbial metabolism. Carbon substrates supplied by biochar and ROF boost the proliferation of functionally specific bacteria. The metabolites secreted by these microbes react with Cd^2+^ to form insoluble precipitates. For instance, sulfate-reducing bacteria reduce SO₄^2−^ to S^2−^, which combines with Cd^2+^ to generate highly insoluble CdS. Moreover, enzymes excreted by microorganisms decompose organic matter in organic fertilizer and release anions that precipitate Cd^2+^. Taking acid phosphatase as an example, it mineralizes organic phosphorus to release phosphate radicals. Extracellular polymeric substances (EPS) produced by microbes directly adsorb free Cd^2+^ via abundant surface functional groups and construct a biological barrier against cadmium mobility. Second, reciprocal interactions between MFA and microorganisms. Acting as electron shuttles, MFA strengthens microbial reductive capacity and facilitates the reduction of soluble Cd^2+^ to elemental Cd (Cd^0^). Meanwhile, MFA synergistically combines microbial metabolites such as S^2−^ and CO₃^2−^ to generate stable Cd-MFA coprecipitates.
(3) Structure–function synergy: Soil amelioration and long-term cadmium sequestration. Firstly, stable soil aggregate formation. Biochar and MFA serve as cementing agents that bind clay particles from organic fertilizer to construct persistent soil aggregates, with Cd encapsulated within aggregate pores and physically isolated from rhizosphere migration. Secondly, coordinated regulation of water retention and redox status. The porous architecture of biochar elevates soil water-holding capacity, which indirectly modulates soil Eh. Under moist paddy conditions, the low-redox microenvironment favors the generation of insoluble CdS, further suppressing Cd bioavailability.

### 4.5 Practical implications, benefits, and field application potential of biochar-based amendment for remediation of mildly cadmium-contaminated paddy soils

Biochar-based composite amendments provide a feasible technical strategy for the safe and sustainable utilization of mildly Cd-contaminated paddy soils. Their agronomic and environmental benefits, rationality of application dosage, economic constraints, and limitations for large-scale field promotion need comprehensive evaluation.

The total application rate of composite amendments was set to 10 t ha^−1^ in the present study. This dosage was determined by summarizing abundant domestic and international field studies focused on the remediation of mildly Cd-contaminated paddy soils, combined with gradient screening results from our pre-experiments (Han et al., 2024). Previous field trials universally recognized 5–15 t ha^−1^ as the practical application range for biochar composite amendments in rice paddies (Payne et al., 2024), and 10 t ha^−1^ balances Cd immobilization efficiency and economic input for smallholder farmers (Xue et al., 2025). Three mixing ratios of biochar (20, 40, and 60%) within composite amendments were arranged to identify the optimal proportion matching biochar, ROF, CMPF, and MFA, which is consistent with the compound amendment design reported by Sun et al. (2023). In terms of field practice, the dosage of 10 t ha^−1^ falls within the widely accepted application range of biochar amendments for farmland heavy metal remediation. One-time basal application before rice transplanting conforms to local agronomic routines in Southwest China without extra complicated farming operations.

In terms of agronomic benefits, converting crop straw into biochar realizes the recycling of agricultural waste. The composite amendments improve soil physicochemical properties, stimulate the activity of soil enzymes involved in nutrient cycling, stabilize bacterial community composition, and alleviate Cd toxicity to soil microorganisms, thereby maintaining soil fertility and supporting sustainable rice production (Ibrahim et al., 2020; Lv et al., 2021). For environmental benefits, the amendments reduce Cd bioavailability via adsorption, ion exchange, and complexation, limiting Cd translocation from soil to rice grains (Li et al., 2019; Xiong et al., 2019). Meanwhile, biochar facilitates soil carbon sequestration to enhance soil carbon sinks. Compared with pure chemical amendments, this organic–inorganic composite material imposes less disturbance on soil ecological functions. In the long run, biochar aging continuously increases soil cation exchange capacity and organic matter content, favoring the persistent immobilization of heavy metals (Chen et al., 2022; Wang et al., 2021).

Regarding economic feasibility and field applicability, raw materials of the amendments are locally available agricultural straw combined with conventional fertilizers, lowering material costs compared with purified commercial biochar (Ndoung et al., 2021). Nevertheless, the one-off application rate of 10 t ha^−1^ adopted in this study creates cost pressure for smallholder farmers. Further research should explore optimized reduced dosage and split application strategies to cut input costs (Nguyen et al., 2023). In field operations, the amendments can be uniformly spread and incorporated into topsoil before rice transplanting, matching conventional tillage practices for rice cultivation in Southwest China without special farming machinery. However, centralized production, transportation, and on-site mixing of composite amendments remain obstacles for extensive promotion. Regional distributed biochar production based on farm straw resources can improve practical operability. When evaluating promotion prospects, ecological benefits and grain safety derived from reduced Cd accumulation in rice should be considered together with direct material costs.

### 4.6 Limitations and future research perspectives

The main limitations of the current study. Biochar can induce persistent long-term alterations to soil physicochemical characteristics, cadmium immobilization efficiency, and soil microbial communities, yet the present field trial only lasted for a single rice-growing season. Consequently, the acquired results only reflect the short-term responses of soil microecology to composite biochar amendments, and it is impossible to judge whether the positive effects on soil enzyme activity, microbial community structure, and Cd bioavailability can be stably maintained in subsequent planting seasons. Additionally, biochar aging may gradually weaken its heavy metal remediation capacity, which cannot be tracked without multi-year continuous monitoring. Several other inherent limitations exist in this article. Firstly, the experimental plots were dismantled after rice harvest for local high-standard farmland renovation, making it impossible to collect long-term dynamic data about soil microecology and Cd immobilization performance during biochar aging. Secondly, only bulk soil samples were collected in this study without rhizosphere soil separation, failing to reveal the microenvironmental variations around rice roots where Cd transformation and microbial metabolism are most intense. Third, sample loss and plot demolition prevented us from carrying out CFU counting of culturable microorganisms and XPS/XRD spectral characterization of aged biochar, lacking direct evidence about cultivable microbe abundance and changes in surface crystal structures or element valence states of biochar. Fourth, the experiment was only conducted on purple paddy soils, without parallel treatments of upland or vegetable soils; thus, the research conclusions are only applicable to analogous rice cultivation regions in Southwest China.

Confounding factors affecting the interpretation of experimental results. Firstly, Intrinsic spatial heterogeneity of field soil, tiny natural differences in initial soil pH, total Cd concentration, and nutrient contents among individual plots inevitably disturb the comparison of passivation performance across treatments. Secondly, Alternating flooding and drying cycles in paddy soils, periodic shifts in soil Eh driven by water management, dynamically regulate Cd speciation transformation and the succession of soil microbial communities. Third, Rice root exudates, root secretions modify local rhizosphere pH and organic matter levels, indirectly altering Cd bioavailability and regulating microbial proliferation. Fourth, Interannual meteorological variation, fluctuations in seasonal rainfall and temperature affect soil moisture, nutrient mineralization rates, and overall soil microbial activity throughout the rice growth cycle.

Future research directions. To address the above limitations, further targeted research is required. Long-term fixed-position field trials in consecutive rice seasons should be carried out to continuously monitor biochar aging processes, dynamic variations in soil Cd fractions, and the stability of microbial regulation effects, so as to systematically verify the sustainability of the remediation benefits observed in this single-season experiment. Meanwhile, rhizosphere soil separation sampling technology, CFU microbial culture assays, and XPS/XRD spectral analysis should be adopted to supplement the missing microecological and material characterization data. Furthermore, multi-type soil parallel experiments covering paddy, upland, and vegetable fields can be arranged to expand the applicability scope of the composite biochar passivator developed in this study.

## 5 Conclusion

This field study demonstrated that biochar-based composite amendments altered paddy soils’ physicochemical traits, which further reshaped soil enzyme activities, microbial biomass pools, bacterial richness, community composition, co-occurrence networks, and metabolic functions. Biochar-based amendments significantly increased soil pH, TN, TP, CEC, SOC, and DOC, while simultaneously decreasing Eh and DTPA-Cd concentration. High-throughput sequencing further confirmed that applied biochar-based amendments increased soil bacterial diversity and abundance, with consequential increasing soil enzyme activity and microbial biomass. PICRUSt analysis revealed that the addition of biochar-based amendments affected amino acid, aromatic compound, carbohydrate, photosynthesis, TCA cycle, glycan biosynthesis and degradation, and other functions. The correlation between soil bacterial community, physiochemical properties, DTPA-Cd content, and rice yield indicated that the favorable effects of biochar-based amendments of DTPA-Cd content and rice yield were mainly attributed to their positive effects on soil physiochemical properties and bacterial community. This research provided a scientific foundation for biochar-based amendments that were effective in alleviating acidified and slightly Cd-contaminated soil in southern China. Nevertheless, since our experimental period was relatively short, the microbial mechanisms of biochar-based amendments application on long-term effects on soil fertility, productivity, and heavy metal Cd passivation need to be further investigated.

## Data Availability

The datasets presented in this study can be found in online repositories. The names of the repository/repositories and accession number(s) can be found in the article/Supplementary material.

## Footnotes

1
https://metacyc.org/

2
https://www.kegg.jp/
