# Supplementary material for: Effects of biochar-based amendments on enzyme activity, microbial biomass, and bacterial communities in mildly cadmium-contaminated paddy soils
Source: Front Microbiol. 2026 Sep 3;17:1923798. doi: 10.3389/fmicb.2026.1923798 (PMC13582494; doi:10.3389/fmicb.2026.1923798)
Supplement: Supplementary file 1 [file Data_sheet_1.DOCX]

**Effects of biochar-based amendments on enzyme activity, microbial biomass, and bacterial communities in mildly cadmium-contaminated paddy soil**

Junnan Han^1,2^, Haining Yang^1^, Baowen Huang^2^, Yuan shi^2^, Wei Peng^1^, Wenjing Zeng^1^, Jian Wang^1^, Donghai Wu^2,3^, Aike Zhu^1*^

^1^Nanchong Academy of Agricultural Sciences, Sichuan 637000, China

^2^Key Laboratory of Plant Hormones and Molecular Breeding of Chongqing, School of Life Sciences, Chongqing University, Chongqing 401331, China

^3^Chongqing Haiyu Environmental Protection Technology Co., Ltd., Chongqing 401122, China

^*^Corresponding author E-mail: aikzhu@126.com

Table S1 Sample sequence quantity.

| Treatment | Input | Filtered | Denoised | Merged | Non-chimeric | Non-singleton |
| --- | --- | --- | --- | --- | --- | --- |
| CK_1 | 89630 | 82615 | 74441 | 46509 | 43897 | 43620 |
| CK_2 | 106779 | 98362 | 91262 | 65673 | 62288 | 62071 |
| CK_3 | 77132 | 71896 | 65649 | 46828 | 44787 | 44607 |
| A1_1 | 87907 | 81778 | 73927 | 47020 | 44203 | 43977 |
| A1_2 | 91450 | 85332 | 78131 | 53392 | 50754 | 50568 |
| A1_3 | 97488 | 89876 | 83295 | 59421 | 56503 | 56309 |
| A2_1 | 88653 | 82064 | 74771 | 49476 | 47037 | 46801 |
| A2_2 | 72667 | 66821 | 60246 | 39921 | 37555 | 37367 |
| A2_3 | 70839 | 65713 | 59338 | 38961 | 37056 | 36860 |
| A3_1 | 84253 | 77661 | 70656 | 46252 | 43551 | 43342 |
| A3_2 | 72495 | 67340 | 61105 | 42124 | 39722 | 39547 |
| A3_3 | 61031 | 56378 | 51905 | 33386 | 30842 | 30710 |
| B1_1 | 69345 | 64427 | 58212 | 37373 | 35257 | 35062 |
| B1_2 | 73492 | 68480 | 61578 | 39840 | 37417 | 37203 |
| B1_3 | 55641 | 50975 | 47158 | 32798 | 30282 | 30164 |
| B2_1 | 77545 | 71952 | 65064 | 42288 | 39816 | 39601 |
| B2_2 | 82107 | 75739 | 69102 | 46054 | 43219 | 43008 |
| B2_3 | 61386 | 56601 | 51965 | 33991 | 31466 | 31309 |
| B3_1 | 85304 | 79568 | 72680 | 47317 | 44800 | 44583 |
| B3_2 | 80410 | 74422 | 67290 | 44768 | 42262 | 42069 |
| B3_3 | 57621 | 52993 | 48663 | 30780 | 28233 | 28121 |
| C1_1 | 89952 | 83512 | 76787 | 53326 | 50631 | 50447 |
| C1_2 | 79404 | 73534 | 66984 | 46373 | 43967 | 43776 |
| C1_3 | 60260 | 55934 | 51286 | 33624 | 31098 | 30957 |
| C2_1 | 81427 | 75357 | 69777 | 51170 | 48927 | 48802 |
| C2_2 | 80769 | 74435 | 68548 | 49698 | 47415 | 47286 |
| C2_3 | 57011 | 52270 | 48685 | 33224 | 30781 | 30678 |
| C3_1 | 84793 | 78666 | 72175 | 51403 | 48810 | 48680 |
| C3_2 | 76478 | 70845 | 64343 | 44799 | 42751 | 42571 |
| C3_3 | 73138 | 67651 | 61122 | 41750 | 39669 | 39499 |
| Total | 2326407 | 2153197 | 1966145 | 1329539 | 1254996 | 1249595 |

Table S2 Notes on taxonomy species.

| Treatment | Domain | Phylum | Class | Order | Family | Genus | Species | Average |
| --- | --- | --- | --- | --- | --- | --- | --- | --- |
| CK | 31.67 ± 12.58 | 27.33 ± 4.93 | 65.00 ± 6.56 | 261.33 ± 31.01 | 364.33 ± 26.41 | 1948.33 ± 169.47 | 62.00 ± 7.00 | 394.28 |
| A1 | 28.00 ± 6.24 | 26.00 ± 4.36 | 58.00 ± 4.58 | 254.33 ± 11.02 | 373.67 ± 19.40 | 1970 .00 ± 78.50 | 68.67 ± 5.13 | 396.95 |
| A2 | 23.00 ± 6.56 | 23.67 ± 6.11 | 51.67 ± 7.02 | 248.67 ± 30.55 | 346.67 ± 45.08 | 1740.00 ± 122.50 | 59.33 ± 7.15 | 356.14 |
| A3 | 17.67 ± 5.69 | 25.00 ± 7.00 | 46.33 ± 6.66 | 209.33 ± 53.80 | 312.67 ± 60.54 | 1565.33 ± 248.40 | 51.67 ± 17.79 | 318.29 |
| B1 | 29.00 ± 15.87 | 22.67 ± 7.57 | 41.67 ± 12.66 | 190.67 ± 51.48 | 304.67 ± 54.31 | 1508.33 ± 268.60 | 61.33 ± 13.20 | 308.33 |
| B2 | 22.33 ± 1.15 | 25.33 ± 2.08 | 48.00 ± 6.56 | 230.00 ± 78.25 | 321.33 ± 26.86 | 1587.33 ± 228.13 | 56.67 ± 2.08 | 327.28 |
| B3 | 24.67 ± 8.14 | 19.67 ± 6.11 | 58.33 ± 20.23 | 215.67 ± 63.80 | 322.67 ± 64.47 | 1574.33 ± 368.11 | 58.00 ± 21.00 | 324.76 |
| C1 | 19.67 ± 4.73 | 24.33 ± 3.79 | 47.67 ± 3.51 | 205.67 ± 67.49 | 326.00 ± 39.85 | 1629.67 ± 258.53 | 54.00 ± 10.82 | 329.57 |
| C2 | 15.67 ± 7.51 | 25.00 ± 7.00 | 45.00 ± 2.65 | 185.67 ± 45.21 | 278.67 ± 59.18 | 1534.33 ± 306.41 | 47.33 ± 15.53 | 304.52 |
| C3 | 20.33 ± 6.43 | 29.67 ± 4.16 | 52.00 ± 9.85 | 198.67 ± 16.56 | 348.67 ± 4.73 | 1652.67 ± 27.06 | 67.00 ± 2.65 | 338.43 |

Data are presented as mean ± standard error (n = 3).

Table S3 Number of microbial taxa.

| Treatment | Domain | Phylum | Class | Order | Family | Genus | Species |
| --- | --- | --- | --- | --- | --- | --- | --- |
| CK | 1.00 ± 0.00 | 42.67 ± 3.21 | 120.33 ± 5.03 | 219.00 ± 7.00 | 282.00 ± 9.64 | 335.67 ± 7.57 | 45.67 ± 2.08 |
| A1 | 1.00 ± 0.00 | 43.00 ± 2.00 | 118.33 ± 2.89 | 214.67 ± 5.51 | 278.33 ± 11.59 | 332.67 ± 9.61 | 50.00 ± 5.20 |
| A2 | 1.00 ± 0.00 | 40.67 ± 2.31 | 119.00 ± 4.00 | 212.67 ± 4.16 | 271.33 ± 4.51 | 312.67 ± 8.02 | 44.67 ± 4.62 |
| A3 | 1.00 ± 0.00 | 41.67 ± 3.06 | 114.67 ± 5.51 | 205.67 ± 12.10 | 265.00 ± 27.40 | 313.00 ± 32.42 | 40.33 ± 10.97 |
| B1 | 1.00 ± 0.00 | 38.00 ± 2.00 | 107.67 ± 6.81 | 195.00 ± 16.00 | 251.67 ± 18.88 | 300.67 ± 25.79 | 42.67 ± 8.50 |
| B2 | 1.00 ± 0.00 | 38.67 ± 0.58 | 111.00 ± 3.00 | 201.00 ± 4.36 | 260.00 ± 7.00 | 305.67 ± 12.86 | 40.67 ± 4.93 |
| B3 | 1.00 ± 0.00 | 39.67 ± 3.79 | 111.33 ± 7.09 | 199.67 ± 9.07 | 251.00 ± 15.52 | 292.33 ± 21.55 | 41.00 ± 9.17 |
| C1 | 1.00 ± 0.00 | 41.33 ± 1.15 | 115.00 ± 6.24 | 211.33 ± 8.14 | 271.00 ± 19.08 | 320.33 ± 26.27 | 42.67 ± 11.02 |
| C2 | 1.00 ± 0.00 | 40.67 ± 4.04 | 114.67 ± 10.69 | 212.67 ± 20.65 | 270.00 ± 34.39 | 316.33 ± 37.87 | 37.00 ± 11.53 |
| C3 | 1.00 ± 0.00 | 40.67 ± 2.08 | 118.33 ± 2.89 | 211.33 ± 2.08 | 276.67 ± 8.08 | 329.00 ± 14.42 | 50.33 ± 4.51 |

Data are presented as mean ± standard error (n = 3).

Table S4 The differences in dominant bacterial among different treatments on phylum level.

| Treatment | Proteobacteria | Chloroflexi | Acidobacteriota | Actinobacteriota | Myxococcota | Gemmatimonadota | Desulfobacterota | Nitrospirota | Bacteroidota | Verrucomicrobiota |
| --- | --- | --- | --- | --- | --- | --- | --- | --- | --- | --- |
| CK | 21.52 ± 1.15a | 19.99 ± 2.08a | 16.94 ± 1.26a | 10.32 ± 0.92b | 5.20 ± 0.09a | 4.07 ± 0.7ab | 4.75 ± 0.68ab | 3.67 ± 0.44a | 2.77 ± 0.48bc | 2.57 ± 1.05a |
| A1 | 22.54 ± 1.93a | 21.98 ± 2.30a | 15.39 ± 0.77ab | 11.96 ± 0.20b | 5.02 ± 0.35a | 4.44 ± 0.35ab | 4.21 ± 0.43abc | 2.76 ± 0.23a | 2.43 ± 0.46c | 2.06 ± 0.96a |
| A2 | 21.79 ± 1.95a | 22.41 ± 3.70a | 15.12 ± 0.56abc | 10.50 ± 1.42b | 4.97 ± 0.54a | 4.87 ± 0.40a | 4.47 ± 0.44abc | 3.13 ± 0.29a | 2.72 ± 0.22bc | 2.62 ± 0.39a |
| A3 | 23.46 ± 0.59a | 19.44 ± 2.47a | 14.83 ± 1.65abc | 13.62 ± 3.96ab | 5.15 ± 0.42a | 3.99 ± 0.42b | 4.82 ± 0.72ab | 3.21 ± 0.41a | 2.67 ± 0.34bc | 2.23 ± 0.45a |
| B1 | 23.61 ± 1.96a | 19.80 ± 3.11a | 14.64 ± 0.31abc | 11.48 ± 0.80b | 5.46 ± 0.45a | 4.14 ± 0.44ab | 5.05 ± 0.77a | 3.80 ± 0.46a | 2.57 ± 0.25c | 2.07 ± 0.79a |
| B2 | 23.50 ± 0.60a | 20.85 ± 3.25a | 14.68 ± 1.41abc | 11.67 ± 1.71b | 5.12 ± 0.18a | 4.12 ± 0.11ab | 4.85 ± 0.43ab | 3.35 ± 1.08a | 2.73 ± 0.27bc | 2.03 ± 0.65a |
| B3 | 22.51 ± 1.44a | 21.42 ± 2.58a | 14.89 ± 1.20abc | 11.52 ± 1.26b | 4.83 ± 0.26a | 4.42 ± 0.05ab | 4.62 ± 0.29ab | 3.78 ± 0.17a | 2.27 ± 0.12c | 2.53 ± 0.13a |
| C1 | 23.83 ± 0.58a | 18.14 ± 1.73a | 13.33 ± 1.20bc | 14.2 ± 2.21ab | 5.39 ± 0.26a | 4.31 ± 0.26ab | 3.93 ± 0.75abc | 4.00 ± 0.81a | 3.39 ± 0.22ab | 1.88 ± 0.27a |
| C2 | 23.70 ± 0.65a | 16.50 ± 0.62a | 13.54 ± 0.68bc | 16.74 ± 1.76a | 5.18 ± 0.15a | 4.54 ± 0.33ab | 3.51 ± 0.67bc | 4.04 ± 0.64a | 3.70 ± 0.44a | 1.38 ± 0.49a |
| C3 | 23.73 ± 0.41a | 18.64 ± 1.98a | 12.69 ± 0.39c | 14.90 ± 2.84ab | 4.76 ± 0.35a | 4.55 ± 0.08ab | 3.21 ± 0.36c | 3.42 ± 0.53a | 4.03 ± 0.32a | 2.12 ± 0.20a |

Data are presented as mean ± standard error (n = 3). Same lowercase letters mean no significant difference while different lowercase letters mean significant differences between groups (ANOVA, LSD test, p < 0.05).

Table S5 The differences in dominant bacterial among different treatments on genus level.

| Treatment | RBG-13-54-9 | SBR1031 | KD4-96 | Thiobacillus | Anaeromyxobacter | SC-I-84 | Subgroup_7 | Anaerolinea | Sideroxydans | Subgroup_17 |
| --- | --- | --- | --- | --- | --- | --- | --- | --- | --- | --- |
| CK | 4.95 ± 0.68ab | 3.38 ± 0.40a | 2.78 ± 0.11a | 0.99 ± 0.44b | 2.28 ± 0.05ab | 2.45 ± 0.24a | 2.22 ± 0.46a | 1.31 ± 0.83a | 1.32 ± 0.43a | 1.85 ± 0.23a |
| A1 | 4.97 ± 0.66ab | 3.82 ± 0.89a | 2.78 ± 0.26a | 2.25 ± 0.79ab | 2.23 ± 0.23ab | 2.01 ± 0.1ab | 1.95 ± 0.24a | 1.94 ± 1.23a | 1.54 ± 0.15a | 1.50 ± 0.32abc |
| A2 | 5.47 ± 0.57a | 3.27 ± 0.35a | 2.66 ± 0.09a | 2.00 ± 0.42ab | 2.2 ± 0.21ab | 2.02 ± 0.15ab | 1.92 ± 0.07a | 1.85 ± 0.47a | 1.48 ± 0.44a | 1.47 ± 0.15abc |
| A3 | 4.86 ± 0.90ab | 3.26 ± 0.78a | 2.65 ± 0.29a | 3.05 ± 0.52a | 2.24 ± 0.30ab | 2.10 ± 0.26ab | 1.82 ± 0.26a | 1.41 ± 0.23a | 1.73 ± 0.63a | 1.71 ± 0.07ab |
| B1 | 4.77 ± 0.61ab | 3.08 ± 0.34a | 2.56 ± 0.18a | 2.76 ± 1.01a | 2.47 ± 0.17a | 2.11 ± 0.20ab | 1.79 ± 0.25a | 1.88 ± 0.90a | 1.81 ± 0.56a | 1.65 ± 0.23ab |
| B2 | 5.16 ± 0.09ab | 3.05 ± 0.61a | 2.47 ± 0.30a | 3.20 ± 0.63a | 2.36 ± 0.17ab | 2.16 ± 0.15ab | 1.79 ± 0.33a | 2.03 ± 1.03a | 1.57 ± 0.22a | 1.45 ± 0.25abc |
| B3 | 4.79 ± 0.39ab | 3.30 ± 0.85a | 2.65 ± 0.11a | 2.06 ± 0.73ab | 1.99 ± 0.11b | 2.21 ± 0.29ab | 1.96 ± 0.25a | 1.90 ± 0.39a | 1.63 ± 0.24a | 1.65 ± 0.1ab |
| C1 | 4.26 ± 0.13ab | 2.99 ± 0.33a | 2.25 ± 0.33a | 2.17 ± 0.61ab | 2.25 ± 0.15ab | 2.03 ± 0.23ab | 1.89 ± 0.29a | 1.33 ± 0.36a | 1.78 ± 0.42a | 1.45 ± 0.23abc |
| C2 | 4.14 ± 0.52b | 3.39 ± 0.24a | 2.53 ± 0.36a | 2.07 ± 0.49ab | 1.98 ± 0.07b | 1.98 ± 0.08b | 2.07 ± 0.04a | 0.91 ± 0.25a | 1.48 ± 0.20a | 1.37 ± 0.06bc |
| C3 | 4.26 ± 0.20ab | 2.75 ± 0.43a | 2.23 ± 0.17a | 1.95 ± 0.09ab | 2.00 ± 0.23b | 2.39 ± 0.02a | 2.22 ± 0.15a | 1.59 ± 0.21a | 1.42 ± 0.32a | 1.19 ± 0.06c |

Data are presented as mean ± standard error (n = 3). Same lowercase letters mean no significant difference while different lowercase letters mean significant differences between groups (ANOVA, LSD test, p < 0.05).

Fig. S1. (a) Notes on taxonomy species, (b) Statistics on number of classified units.

Fig. S2. Taxonomic Tree in Packed Circles (a). Taxonomic differences are based on 16S rRNA, sequences extracted from the metagenome. The largest circles represent phylum level, and the inner circles represent class, family, and genus. Phylogenetic tree of genus level during biochar-based amendment soil (b).

Fig. S3. Rarefaction curves of the observed species (a), Shannon index (b), and rank abundance curve (c).

Fig. S4. Principal coordinates analysis (PCoA) of the bacterial community for different treatments.

Fig. S5. Species taxonomic loadings.
